# A market and risk assessment of 125 turmeric supplements available in Australia, Germany, India, UK, and USA

**DOI:** 10.1007/s00210-025-04392-5

**Published:** 2025-08-07

**Authors:** Haleema Rahim-Mahdy, Roland Seifert

**Affiliations:** Institute of Pharmacology, Hannover Medical School, Carl-Neuberg-Straße 1, D-30625 Hannover, Germany

**Keywords:** Bioavailability, *Curcuma longa*, Curcuminoids, Detoxification, Drug metabolism, Labeling, Nanoparticles, Oxidative stress, Pro-oxidant, Regulation, Safety, Supplement, Therapeutic doses, Toxicity, Turmeric

## Abstract

Historically prominent in Ayurvedic cultures, turmeric (*Curcuma longa*), or “Haldi,” is renowned for its anti-inflammatory and antioxidant effects, mainly due to its active compounds, curcuminoids. Due to the increasing use of turmeric supplements, this study critically examined 125 preparations across the UK, USA, India, Australia, and Germany and evaluated their compliance with evidence-based recommendations. Results reveal significant regulatory and labeling inconsistencies across countries, with 34% of preparations failing to disclose the active curcuminoid content. This lack of dosage regulation is especially concerning when considering the ongoing research into advanced delivery systems—such as nanoparticles, liposomes, micelles, and phospholipid complexes—which significantly enhance curcumin’s absorption. Curcumin, a hydrophobic compound, undergoes rapid metabolism in the liver through Phase I and II detoxification pathways, particularly via cytochrome P450 enzymes and UDP-glucuronosyltransferase enzymes. This results in limited bioavailability, as curcumin is rapidly converted to water-soluble metabolites and excreted, reducing its effectiveness at therapeutic doses. However, through modern formulation technologies, curcumin can potentially not only alter drug metabolism, but its antioxidant action via Nrf2 activation can shift to pro-oxidant effects at high doses, causing oxidative stress and the accumulation of reactive metabolites. Emerging evidence suggests chronic low-dose use may lead to gastrointestinal, hepatic, or renal toxicity, yet turmeric preparations, falling within the scope of food law, lack the stringent controls applied to pharmaceuticals and are generally assumed safe. This study highlights the need for transparent labeling, clear dosage guidelines, and an understanding of curcumin’s metabolic profile to guide consumers in maximizing benefits while mitigating risks. Furthermore, it aims to assist healthcare professionals in making informed recommendations regarding curcumin supplementation. Further research must include long-term clinical trials and potential standardization of curcumin supplement formulations.

**Supplementary Information:**

The online version contains supplementary material available at 10.1007/s00210-025-04392-5.

## Introduction

Turmeric, a member of the ginger Zingiberaceae family, has deep roots in Ayurvedic culture, dating back as far as 4000 years ago in South Asia where it is natively cultivated (Dei Cas et al. 2019, Ahmad et al. 2020). Belonging to the genus Curcuma, this plant includes over 100 species, with *Curcuma longa* being the most widely recognized and cultivated. However, there is considerable intraspecific variability, with each species exhibiting differences in bioactive compound profiles. Some of these variations are summarized in Table 1, though this list is not exhaustive (Yuandani et al. 2021). Traditionally used in cooking and dyes, turmeric’s medicinal value stems from its curcuminoids—a group of bioactive compounds including curcumin, demethoxycurcumin, and bis-demethoxycurcumin, found in the plants’ rhizomes (Dei Cas et al. 2019). Turmeric contains 2–5% curcuminoids by weight, with curcumin, or diferuloylmethane, constituting approximately 77% of the total curcuminoid content, while demethoxycurcumin and bis-demethoxycurcumin account for 18% and 5%, respectively. Its constituents are further discussed in Table 2, with variable percentages depending on the extraction method.

Curcumin is a hydrophobic polyphenol diarylheptanoid, consisting of two aromatic ring systems, each substituted with hydroxyl and methoxy groups, linked by a seven-carbon chain containing a β-diketone moiety (Abd El-Hack et al. 2021). This structure provides curcumin with the ability to undergo keto-enol tautomerism, existing predominantly in the enol form in solution. The system of conjugated double bonds also facilitates its strong antioxidant properties by allowing it to donate electrons and neutralize free radicals. The phenolic and methoxy groups on the aromatic rings enhance curcumin’s radical-scavenging activity and its ability to chelate metal ions to catalyze the generation of reactive oxygen species. Additionally, the β-diketone structure is critical for curcumin’s interaction with various molecular targets, such as enzymes and transcription factors. For instance, it binds to thiol groups in proteins, modulating signaling pathways like NF-κB and Nrf2, which upregulate cellular defense mechanisms like glutathione synthesis. This supports Ayurveda’s emphasis on turmeric for detoxification and vitality enhancement. Its anti-inflammatory action, mediated by the inhibition of NF-κB and COX-2 pathways, aligns with its traditional use in treating inflammatory conditions and wounds. Furthermore, curcumin stimulates bile production and enhances lipid metabolism, which explains its Ayurvedic application for digestive disorders

**Table 1:** Variations in Curcuma species

Species	Common names	Origin and distribution	Curcumin content	Uses in traditional medicine	Appearance
*Curcuma longa*	Haridra (Sanskrit, Ayurvedic), Jianghuang (Chinese), Kyoo or Ukon (Japanese), Kurkum (Arabic), and Haldi (Hindi and Urdu)	South Asia, widespread in tropical areas	2–5%	Antioxidant, anti-inflammatory, antidiabetic, hepatoprotective, antimicrobial	Upright plant, yellow-white flowers (10–15 cm stalk length), brown small ovoid seeds
*Curcumin zanthorrhiza*	Temu Lawak, Java turmeric	Indonesia, Southeast Asia	Low	Anti-inflammatory, anticancer, lowers cholesterol, boosts immunity	Erect pseudostems (2 m tall), rhizomes smell balmy, taste bitter
*Curcuma amada*	Mango ginger	Myanmar, India, tropics	1–2%	Inflammation, asthma, bronchitis, digestive issues, skin diseases	Fleshy rhizomes (5–10 cm long, 2–5 cm diameter), buff-colored
*Curcuma aeruginosa*	Kali Haldi (black turmeric), Temu Ireng	Myanmar, Southeast Asia	Low	Immunity booster, gastrointestinal issues, antimicrobial, anti-inflammatory	Deep-blue or bluish-black rhizomes, pungent odor, perennial
*Curcuma zedoaria*	White turmeric, Ezhu	Bangladesh, India, China	Low	Cancer treatment, blood circulation, flatulent colic, liver issues	Rhizomes with dark orange flesh, 8–12 cm long, tuber-like
*Curcuma mangga*	Mango turmeric	Java, Southeast Asia	Low	Stomach disorders, fever, cancer-related diseases	Perennial herb (30–110 cm tall), rhizomes with mango-like smell

Adapted from Yadav et al. 2017 ), European Medicines Agency 2018), and Kotha et al. 2019 ).

**Table 2.**
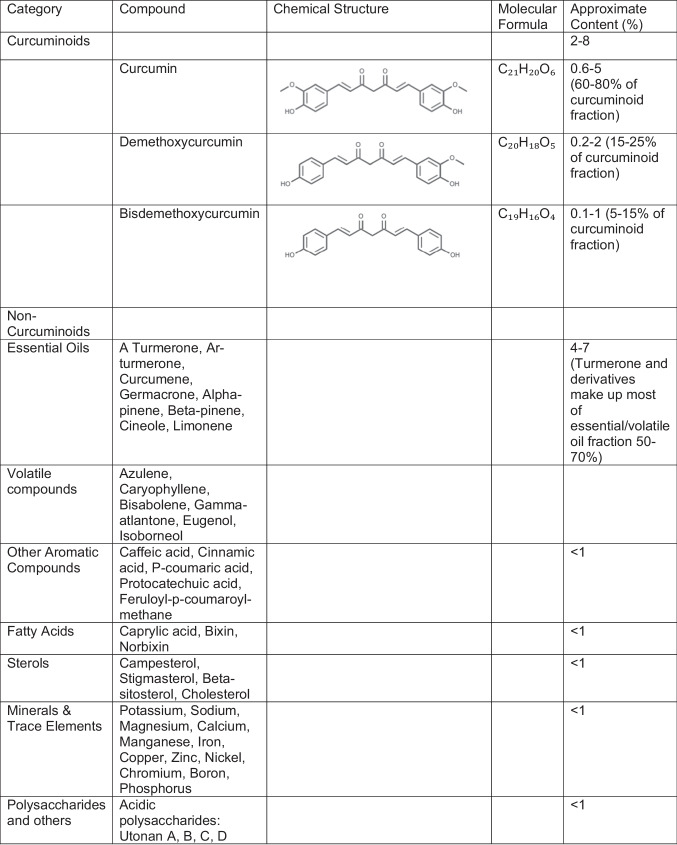
Phytochemicals in *Curcuma longa* rhizome

**Fig. 1:**
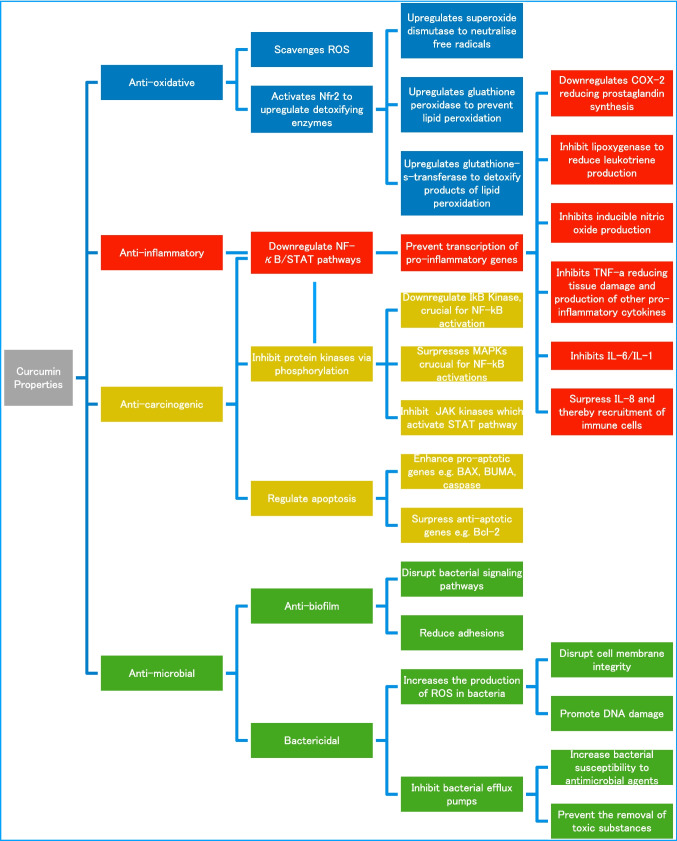
Diagram showing curcumin properties. Adapted from Sohn et al. (2021). The diagram employs a color-coded scheme to summarize the pharmacological properties of curcumin: blue represents its anti-oxidative effects, red denotes anti-inflammatory actions, yellow highlights anti-carcinogenic mechanisms, and green encompasses its anti-microbial functions (including anti-biofilm and bactericidal activity)

Studies have also shown curcumin’s anti-inflammatory, anti-oxidative, anti-carcinogenic, and antimicrobial properties, establishing its role in modern therapeutic research (Sohn et al. 2021). The transcription factor NF-κB regulates genes like cyclin D1, p21, MMP-1, and MMP-3, which contribute to cancer cell growth and survival. Chronic NF-κB activation inhibits apoptosis, aiding cancer progression. Research showed curcumin reduces breast cancer proliferation by downregulating NF-κB-induced genes, as well as inhibiting the STAT3 transcription factor responsible for cancer cell survival and metastasis in HCC and myeloma (Sohn et al. 2021)). Curcumin targets multiple cancer-related kinases, such as AMP-kinase, enhancing phosphorylation to reduce tumor growth and metastasis in breast cancer models. By enhancing pro-apoptotic genes such as Bax and PUMA and suppressing anti-apoptotic ones like Bcl-2, curcumin effectively induces apoptosis in various cancer cell lines, including hepatoma, mammary epithelial, and breast cancer cells. Curcumin’s anti-inflammatory effects include scavenging ROS by donating hydrogen atoms or electrons from its phenolic and β-diketone groups, which reduces oxidative stress. It also inhibits the expression of inflammatory enzymes such as COX-2, lipoxygenase, and inducible nitric oxide synthase and decreases cytokine production. Furthermore, it downregulates the production of pro-inflammatory cytokines, including TNF-α, IL-1β, IL-6, and IFN-γ, which play critical roles in cancer progression and chronic inflammation. Curcumin acts on both planktonic cells and bacterial biofilms by increasing the production of ROS which disrupts cell membrane integrity and promotes DNA damage. In addition, curcumin inhibits bacterial efflux pumps, preventing the removal of toxic substances and increasing bacterial susceptibility to antimicrobial agents. It also interferes with the formation of biofilms by disrupting bacterial signaling pathways and reducing adhesion, thereby preventing biofilm formation. Figure 1 summarizes the effects of curcumin (Sohn et al. 2021).

The metabolism of curcumin occurs primarily in the liver and small intestine via 2 phases, as well as an alternative pathway using the gut microbiota (Dei Cas et al. 2019). Phase 1 compromises a series of reduction reactions, whereas during Phase 2, the metabolites undergo conjugation. In Phase 1, the reduction of curcumin is catalyzed by various reductase enzymes, including NADPH-dependent reductases found in the liver and gut microbiota, nitroreductases, and azoreductases expressed by several gut bacterial strains, and carbonyl reductase (CR) and aldo–keto reductases (AKRs) in the liver and extrahepatic tissues. Additionally, cytochrome P450 enzymes (CYP) contribute to the oxidative metabolism of curcumin, producing intermediates like hydroxycurcumin and dihydrocurcumin. A key reductase in the reduction process is encoded by the curA gene, identified in bacterial strains such as *Escherichia coli* (Hassaninasab et al., 2011). This curA-mediated reduction involves stepwise hydrogenation of curcumin’s bis-α,β-unsaturated ketone structure, primarily in the gastrointestinal tract, leading to the formation of metabolites such as dihydrocurcumin, tetrahydrocurcumin, and hexahydrocurcumin. The efficiency of curA-mediated reduction and CYP-mediated oxidation is significantly influenced by the composition of an individual’s gut microbiota. Variations in the presence or absence of bacterial strains carrying the curA gene and the activity of specific CYP enzymes contribute to considerable inter-individual differences in curcumin metabolism and therapeutic responses. In Phase II, curcumin undergoes extensive glucuronidation, mediated by UDP-glucuronosyltransferases (UGTs), primarily in the liver and intestines. UGTs catalyze the transfer of glucuronic acid from the cofactor UDP-glucuronic acid to the hydroxyl groups on the curcumin molecule, forming water-soluble glucuronides. This modification facilitates the excretion of curcumin metabolites via bile and urine, but it also significantly reduces the systemic bioavailability of free curcumin, limiting its therapeutic potential. Certain bacterial strains possess enzymatic capabilities, such as β-glucuronidase activity, to deconjugate glucuronides back into free curcumin, enabling reabsorption and prolonging its activity. Strategies to enhance curcumin bioavailability often involve co-administration with UGT inhibitors, such as piperine, which slows glucuronidation and prolongs systemic circulation. Similarly, sulfation represents another key phase II metabolic pathway. Sulfotransferases (SULTs), particularly SULT1A1 and SULT1A3 isoforms, catalyze the transfer of a sulfonate group from the cofactor 3′-phosphoadenosine-5′-phosphosulfate (PAPS) to the hydroxyl groups on curcumin, producing curcumin sulfates. These metabolites are also highly water-soluble and ultimately excreted in the feces and urine (Dei Cas et al. 2019, Wang et al. 2021).

Poor water solubility and the oral bioavailability of a mere 1%, combined with poor absorption, rapid metabolism, and rapid elimination, have ultimately formed a poor pharmacokinetic profile. These factors have proven problematic in introducing curcuminoids into clinical application, significantly limiting its pharmaceutical benefit (Yang et al. 2007, Wang et al. 2021, Hewlings et al. 2017). Some supplements incorporate substances designed to enhance curcumin’s bioavailability. For example, piperine, the active component of black pepper, has been shown to increase curcumin’s bioavailability by up to 2000% (Hewlings et al. 2017). Other advanced formulations, such as nanocurcumin and liposomal encapsulation, modify various aspects of curcumin’s metabolic pathway to improve absorption and therapeutic effectiveness (Hewlings et al. 2017). Nanoparticles, including polymeric nanoparticles, solid lipid nanoparticle, and nanocrystals, increase the surface area of curcumin, promoting better dissolution and absorption in the gastrointestinal tract while also offering sustained release and protection from degradation. Liposomal encapsulation utilizes phospholipid-based vesicles to enhance solubility, stability, and targeted delivery, thereby improving curcumin’s therapeutic efficacy. Similarly, micellar systems, composed of surfactant-formed spherical structures, encapsulate curcumin in their hydrophobic core, significantly enhancing its solubility in water and overall bioavailability. Hydrogels, with their 3D polymeric networks, encapsulate curcumin for controlled release, stability, and sustained or localized delivery. Curcumin-phospholipid complexes combine curcumin with phospholipids to enhance solubility, protect it from degradation, and facilitate better absorption. Lastly, nanoemulsions, composed of nanoscale oil droplets dispersed in water, improve curcumin’s solubility and promote effective absorption (Sohn et al. 2021). 

**Fig. 2:**
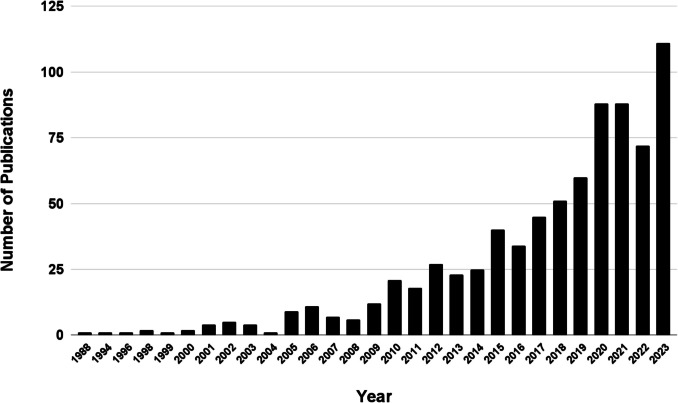
Time trend of publications for “Curcumin Supplements” on PubMed.gov (accessed on 17.08.2024) https://pubmed.ncbi.nlm.nih.gov/?term=curcumin+supplement

**Fig. 3:**
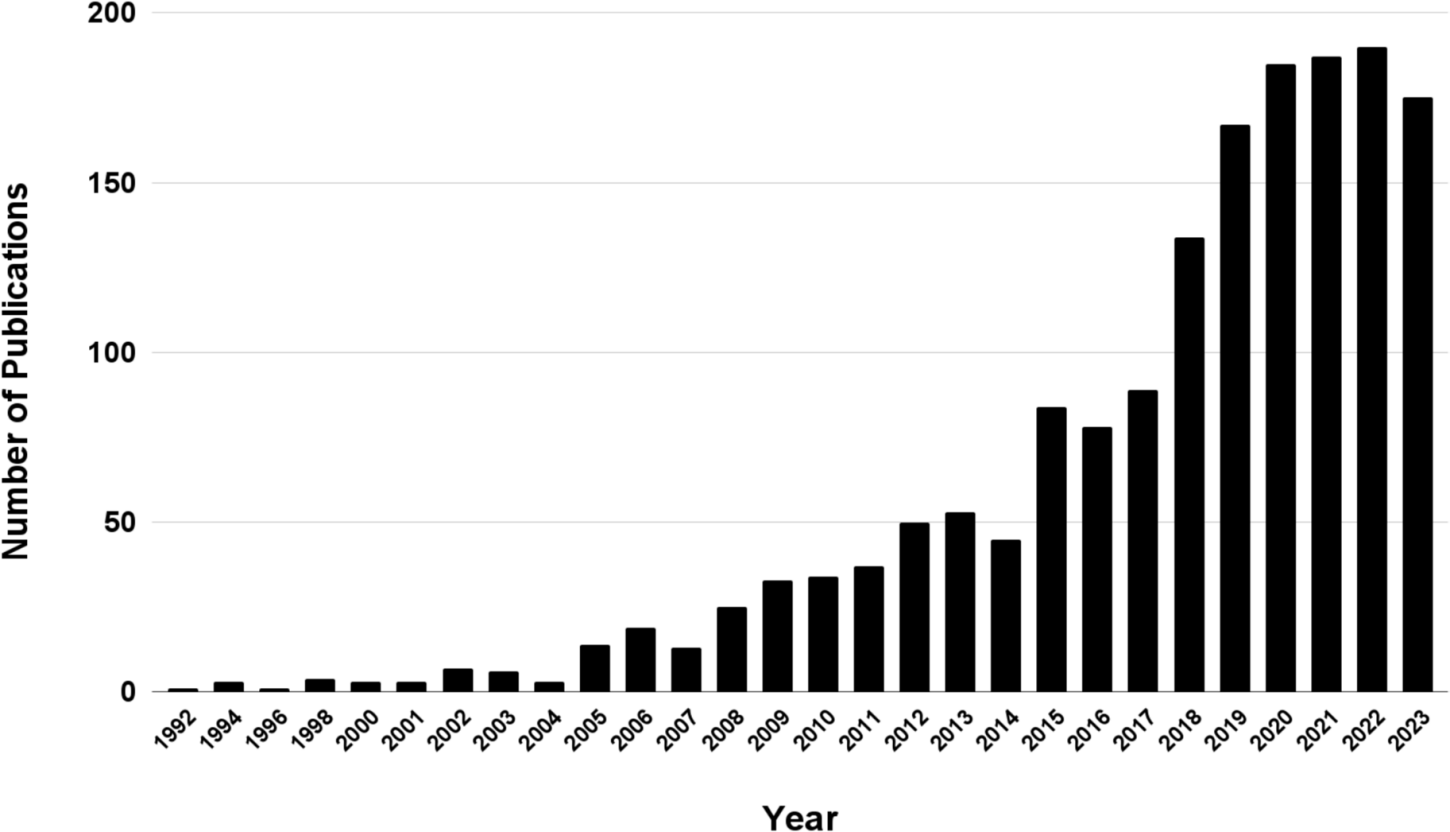
Time trend of publications for “Turmeric Supplements” on pubmed.gov (accessed on 17.08.2024) https://pubmed.ncbi.nlm.nih.gov/?term=turmeric+supplement)

**Fig. 4:**
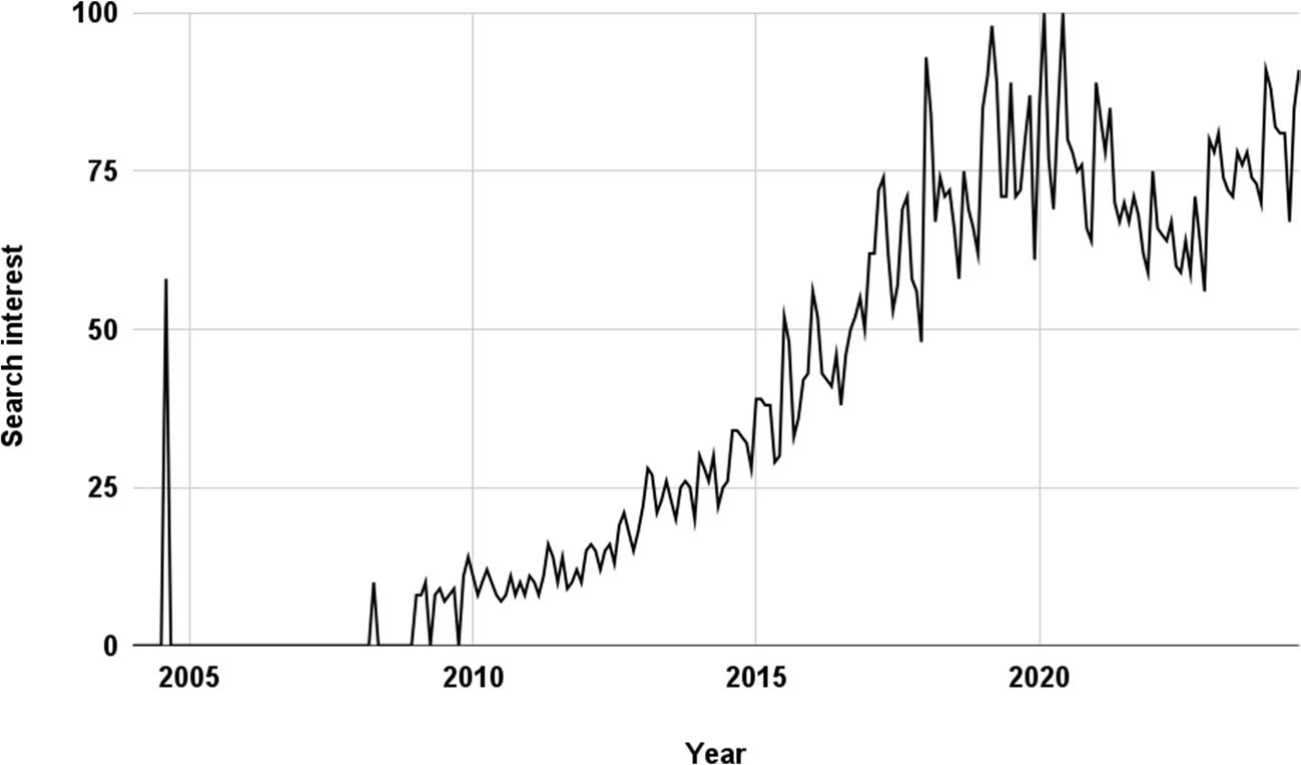
Google search interest since 2004 for “turmeric supplements.” The vertical axis indicates search interest relative to the peak value on the chart for a specific region and time period. A value of 100 represents the highest popularity for the term. A value of 50 means the term is half as popular as the peak. A score of 0 indicates insufficient data for the term. https://trends.google.com/trends/explore?date=all&q=Turmeric%20supplements&hl=en-US (accessed on 17.08.2024)

Research on curcumin and its health benefits has generally been on the rise, paralleling growing public interest (Khosravi and Seifert 2023). Figures 2 and 3 show the increase in publications on PubMed relating to curcumin and turmeric supplements since 1992 and 1988, respectively. Figure 4 represents Google search interest since 2004 for “turmeric supplements.”

The multitude of formulations has led to an influx of turmeric supplements offering differing concentrations of curcuminoids which are readily available to the general public. An increase in liver injury due to turmeric supplementation was suggested in a study conducted from 2004 to 2022, deducing the addition of piperine as an integral factor increasing toxicity (Halegoua-DeMarzio et al. 2023). Although JEFCA established an ADI for curcumin of 0–3 mg/kg in 2004, encompassing food additives, spices, and supplements as sources of curcumin, this recommendation explicitly accounts for curcumin in its natural form and excludes any preparations containing TEES (JEFCA, 2004). This is significant especially when considering highly bioavailable formulations of *Curcuma longa* products were found to be associated with 7 cases of acute hepatitis in Tuscany until September 2019 (Lombardi et al. 2020).

The TGA documented 18 reports of liver injury associated with curcumin supplementation up until 2023, stating the risk could be potentially higher in products with enhanced bioavailability; however, a lack of research in this area means a definitive conclusion cannot be drawn (TGA 2023a, b). The BfR advises individual assessment of supplements with improved bioavailability and potential reduction in the ADI accordingly (Bundesinstitut für Risikobewertung (BfR), 2021), yet curcumin supplementation falls under the regulatory framework of food law in 4 out of 5 countries included in this study, leaving dosage recommendations subject to the influence and bias of manufacturers. In Australia, curcumin is classified as a “complementary medicine,” which similarly lacks the regulation of prescription medication due to being classified as “low-risk” (Ghosh 2014). The absence of formal quality control and corroboration of ingredients, as opposed to the stringent control seen in pharmaceuticals, risks contamination as well as overdose.

A multitude of short-term toxicity studies and research focused on the benefits of curcumin detracts from the absence of concrete evidence for turmeric to be used clinically in the long term (Burgos-Morón et al. 2010). A recent analysis reported no studies showing any evidence of clinical benefit in tumor patients out of 52 publications discussing the use of curcumin in malignant diseases (Khosravi and Seifert 2023). This prompts consideration of the risks and benefits of curcumin supplements, yet consumers are left responsible to independently assess these factors. This is although a survey of 1500 Americans in 2020 showed 75% of supplement users do not research industry certifications before buying (LifeExtension 2020). As a result of a lack of critical evaluation in this area, this study was developed to analyze 125 turmeric supplements freely available to the general public in the UK, USA, Australia, Germany, and India.

## Methods

In total, 125 supplements were analyzed. Twenty-five supplements were analyzed in each of the following countries: UK, USA, Australia, Germany, and India. Package labels of supplements advertised on the respective websites were analyzed in April and May 2022. Table 3 lists all websites used to retrieve products in each country. Table S1 details the preparations further.

The date the information was collected, the website link, the retailer, the ingredients list, and a screenshot of the product were recorded in a table. The label was then analyzed to extract and, when necessary, calculate the following information.

- Type of turmeric used
- Amount of turmeric per unit (mg)
- Recommended units per day
- Turmeric effect enhancing substance (TEES)
- Pack size
- Price per unit (€)

The recommended retail price (RRP) was converted to euros via Google Finance based on the conversion rate on the date the information was collected; this was then divided by the pack size.

g. Advertised benefits
h. Dosage form
i. Overdose warning
j. Warning for use during pregnancy or lactation
k. Indication of interaction with other drugs
l. Indication of adverse effects
m. Indication of target audience

Using this information, the following were calculated.

1. Specified curcuminoid amount per unit (SCA) (mg)
2. Maximum daily dose (MDD) (mg)
3. Cost of maximum daily dose (CMDD) (€)

In some cases, to condense the information and for visualization and comparison purposes, range categories were formed based on distribution of data. The information was then analyzed and compared.

**Table 3:** Retrieval of information of preparations

Country	Website to retrieve data	Date of last product retrieval
Australia	www.chemistwarehouse.com www.barnesnaturals.com www.pharmacyonline.com www.pharmacy4less.com www.priceline.com www.turmericaustralia.com www.pharmacydirect.com www.organicturmeric.com	28.05.2022
UK	www.hollandandbarrett.com www.wildnutrition.com www.cytoplan.co.uk www.amazon.co.uk www.naturesbest.co.uk www.superdrug.co.uk www.boots.com www.ocado.com www.puritanspride.co.uk	29.05.2022
USA	www.target.com www.bioschwartz.com www.walgreens.com	09.05.2022
India	www.pharmeasy.in ww.amazon.in www.1mg.com www.healthkart.com	25.05.2022
Germany	www.shop-apotheke.com www.dm.de	08.05.2022

## Results

### Types of turmeric

**Fig. 5:**
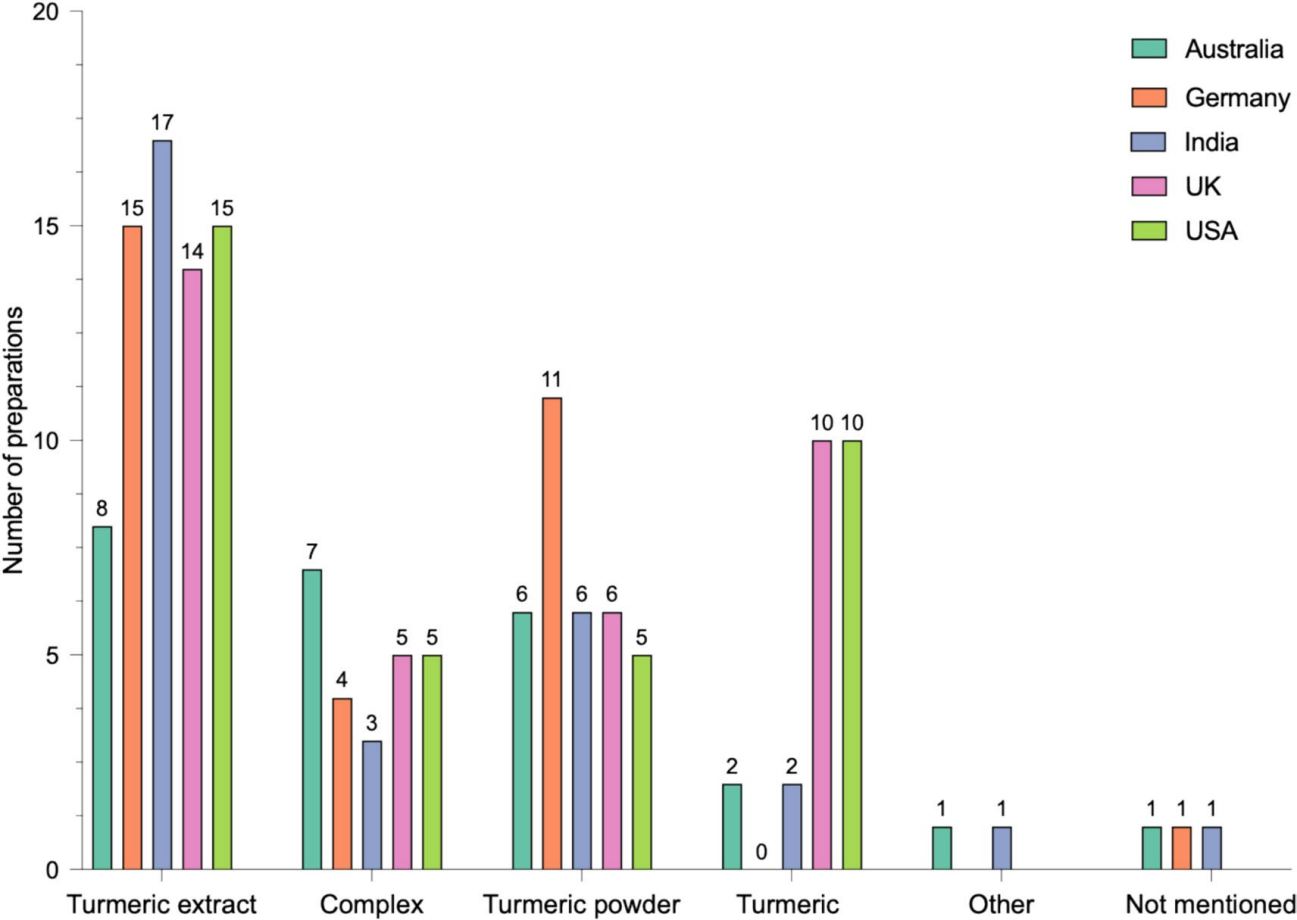
Types of turmeric. Graph showing the frequency of types of turmeric used in supplements in each country. Information is presented in a grouped column diagram, and each country is color-coded

Figure 5 shows the number of preparations in each country using a type of turmeric. Turmeric extract was used most often in all countries. In descending order: in 17/25 preparations in India (68%), 15/25 in Germany and the USA (60%), 14/25 in the UK (56%), and 8/25 in Australia (32%). In total, 3/125 preparations (2.4%) did not mention what type of turmeric was used in the packaging label (1 in Australia, 1 in India, and 1 in Germany). It is worth noting, the product in Australia specified “curcumin, equivalent curcuminoids and rhizome,” the product in Germany specified curcumin and was advertised as a curcumin product rather than a turmeric product, and the product in India specified curcumin with 95% curcuminoids in the ingredients list. The “other” category represents turmeric concentrate being used in 1 preparation in India and an herbal concentrate being used in Australia. Figure S2 details the combinations of turmeric types used in the supplements.

### Turmeric effect enhancing substance (TEES)

**Fig. 6:**
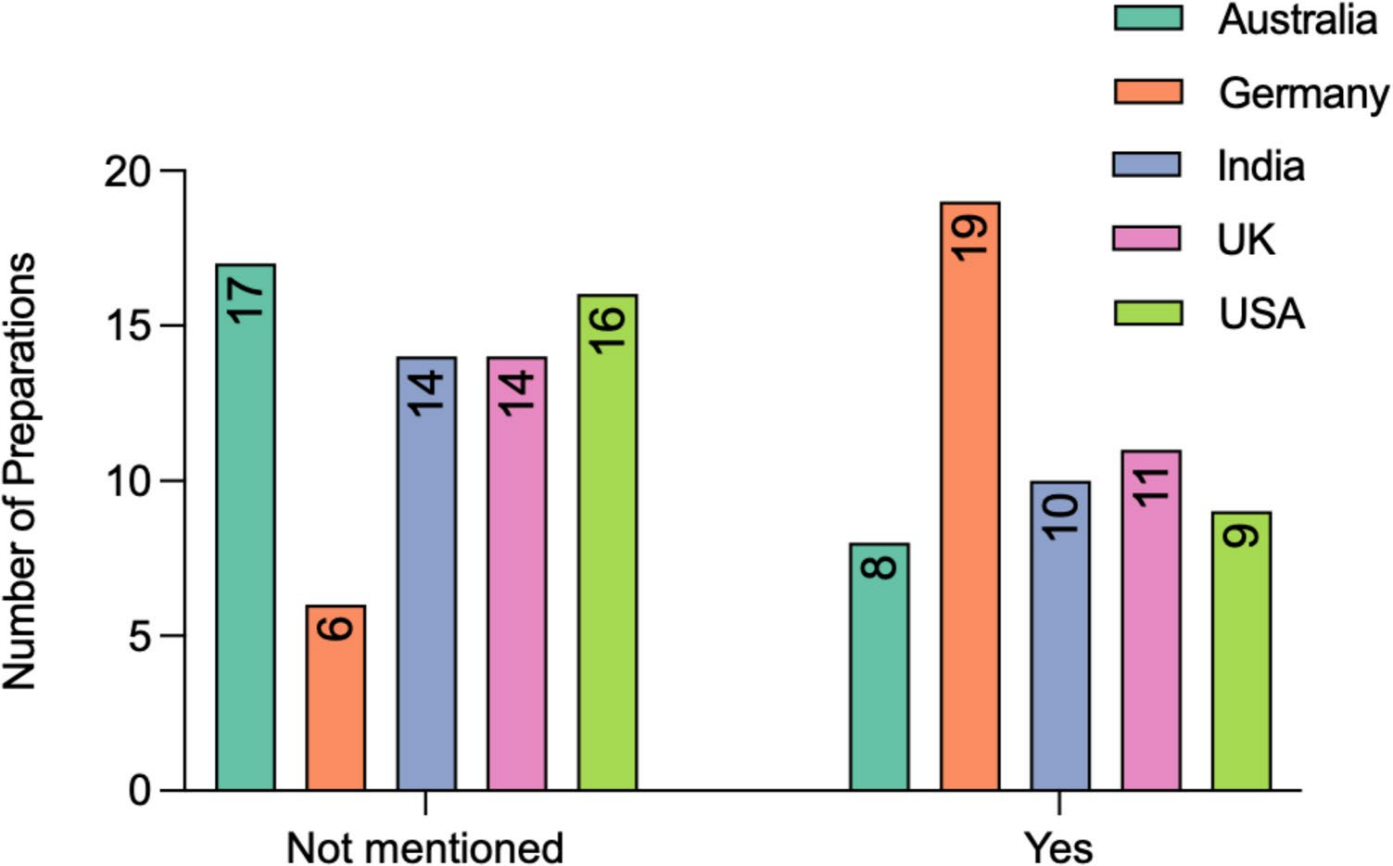
Turmeric effect enhancing substance. Representation of the number of preparations containing a TEES. Information is shown in a bar chart and is color-coded to represent each country

Figure 6 shows the number of preparations per country using, if any, turmeric effect enhancing substances. The majority of preparations (67/125, 53.6%) do not mention any use of a TEES. German preparations most often used a TEES, specifically in 19/25 preparations (76%), followed by the UK with 11/25 preparations (44%), India with 10/25 preparations (40%), and the TEES was most often used by each country. Black pepper extract was the most popular (23/125 preparations, 18.4%). The “other” category includes 1 product in Germany containing long pepper and 1 containing fenugreek, as well as 1 product in India containing a combination of black pepper and long pepper.

Figure S3A, S3B, and S3C shows the number of preparations in each country incorporating a complex with a TEES, the number of preparations in each country using turmeric extract and a TEES, and the number of preparations in each country combining more than 1 type of turmeric with a TEES, respectively.

### Specified curcuminoid amount per unit (SCA) (mg)

**Fig. 7:**
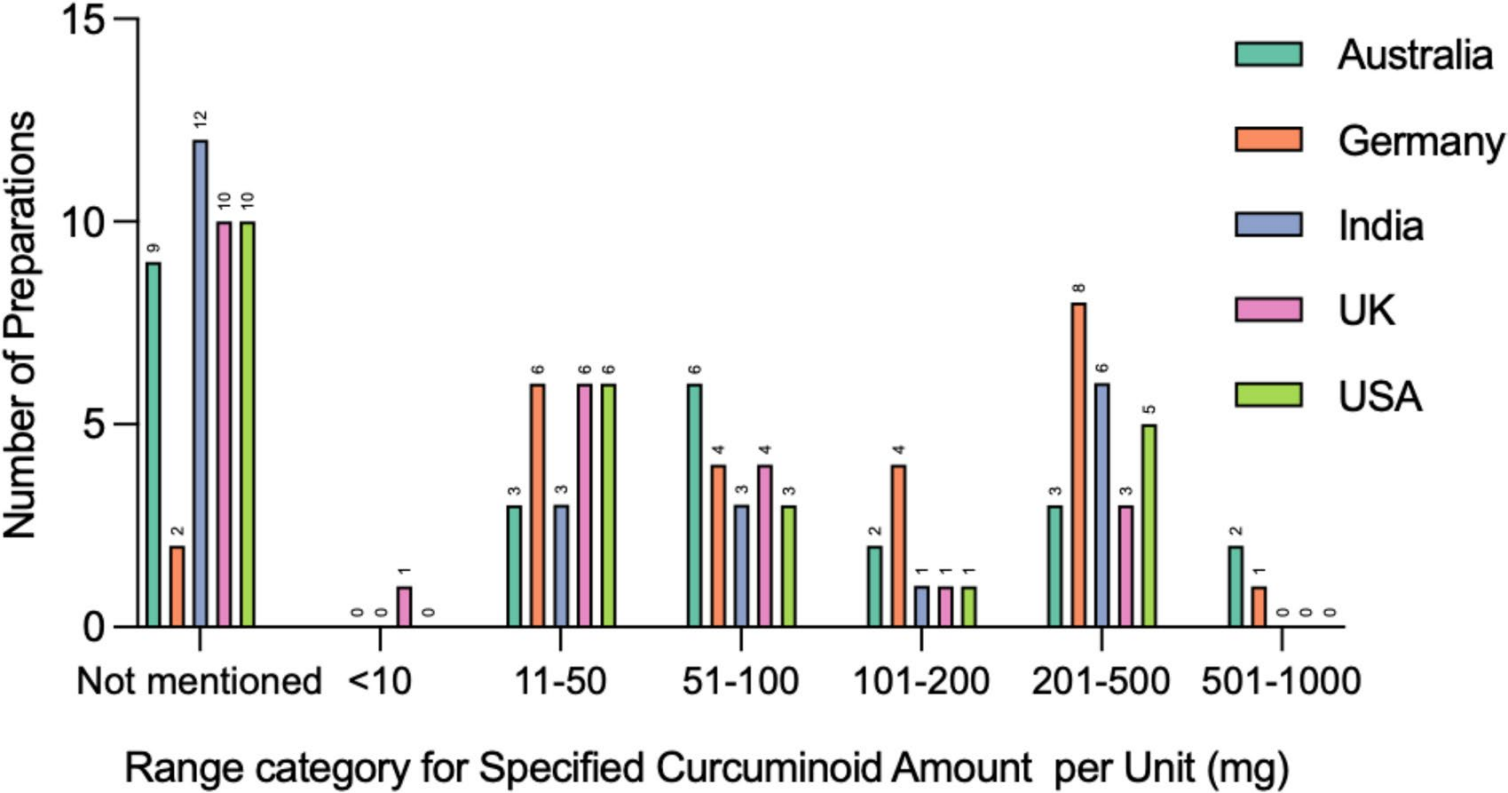
Specified curcuminoid amount per unit (SCA) (mg). Analysis of the specified curcuminoid amount per unit in milligrams. This is represented in a grouped column diagram, showing each country in a different color. The *x*-axis shows range categories from < 10 to 1000 mg

Figure 7 shows the number of preparations per range category for the specific amount of curcuminoids per unit in milligrams. This is the amount of curcuminoids in milligrams that has been specified or able to be deduced from the packaging. Out of the total 125, 43 preparations (34.4%) did not specify an amount, either in percentage form to be calculated or in milligrams. Of these preparations, India had the most preparations (12/25, 48%) where this information could not be identified. A single preparation contained less than 10 mg of curcuminoids per unit; the preparation was from the UK. Most preparations (25/125, 20%) contained between 201 and 500 mg curcuminoids per unit. Australia and Germany contained preparations with the highest amount of curcuminoids per unit (2 preparations and 1 preparation, respectively) between 501 and 1000 mg.

### Maximum daily dose (MDD) (mg)

**Fig. 8:**
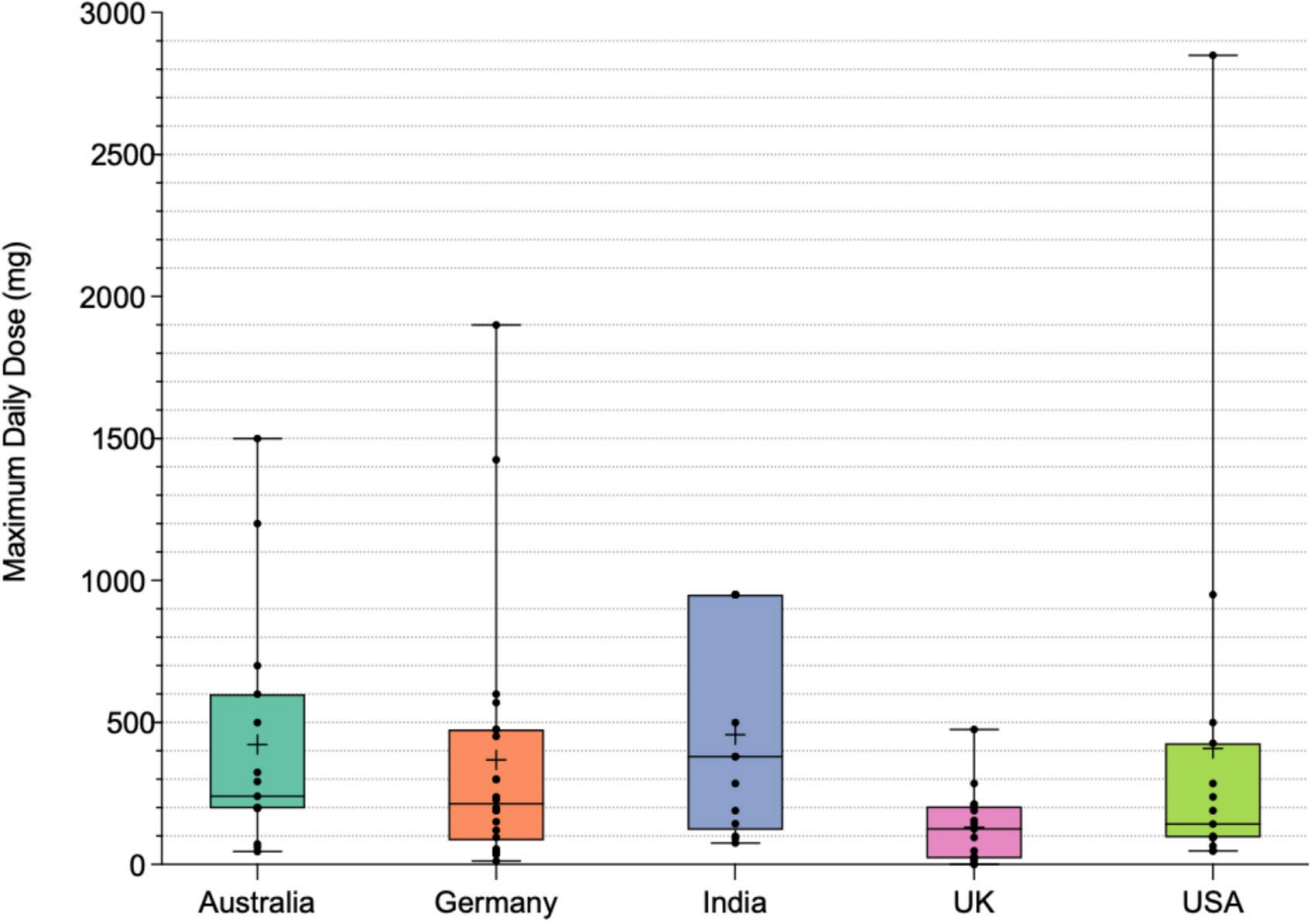
Maximum daily dose (MDD) (mg). Representation of the MDD in a box and whiskers chart. The box extends from the 25th to 75th percentiles, and the median is indicated by the line within the box. The average is denoted by a “ + ”, and the whiskers mark the maximum and minimum values. The dots represent individual values

Figure 8 shows the maximum total dose of curcuminoids recommended per day, as recommended by manufacturers. This was calculated by multiplying the recommended units per day by the specified curcuminoid amount in milligrams per day. In 44/125 preparations (35.2%), this information could not be recorded. Four of the preparations did not recommend a dosage: 2 in Australia and 2 in India. The lowest mean MDD was observed in the UK at 123.4 mg, which is considerably lower than the mean in all other 4 countries, the highest being in India at 457.4 mg. The maximum MDD was observed in the USA, with 1 preparation recommending 2850 mg. This is in stark contrast to the maximum MDD recommended in the UK of 475 mg. The information was available most often in Germany, in 22/25 (88%) of preparations. India, on the other hand, only offered this information in 13/25 (52%) of preparations. 22/125 preparations (17.6%) did not recommend a specific number of units, rather a range; here, the maximum recommendation was used. Table 4 summarizes the mean, median, maximum, and minimum values for each country.

**Table 4:** Summary of statistical values for MDD

	**Mean (mg)**	**Median (mg)**	**Minimim value (mg)**	**Maximum value (mg)**	**Number of preparations**
Australia	422.2	240	46	1500	16
Germany	368	213.8	12	1900	22
India	457.4	380	75.2	950	13
UK	123.4	110	12.5	475	15
USA	408.5	142.5	47.5	2850	17

Figure S4 shows the number of preparations in each country recommending an MDD above and below the maximum dose recommended by JEFCA for an average adult weighing 70 kg, equating to 210 mg (JEFCA, 2004). Each country differs in the number of preparations offering this information. In total, the UK offers the least number of preparations offering a dose higher than the JEFCA recommendation (3/15 preparations, 20%), followed by the USA (6/17 preparations, 35%). Germany offers the most preparations exceeding the JEFCA recommendation (11/25 preparations, 44%).

### Cost of maximum daily dose (CMDD) (€)

**Fig. 9:**
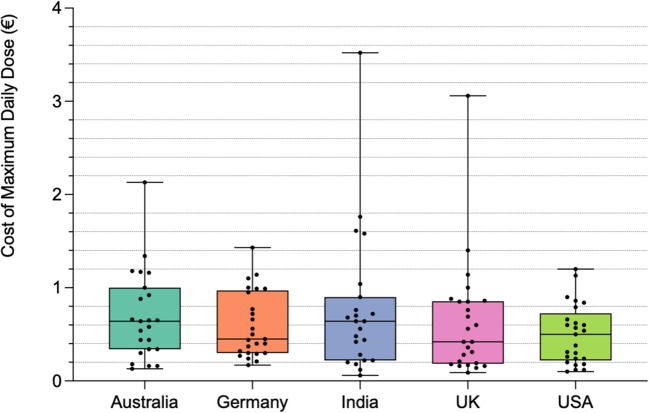
Cost of maximum daily dose (CMDD) (€). Graphic representation of the CMDD (€) as a scatter dot graph. The dots show individual data. The bar corresponds to the median, and the “ + ” indicates the mean. The whiskers correspond to the minimum and maximum value. The countries are color-coded

Figure 9 shows the international and intra-national variation in cost of the maximum daily dose. In 4 preparations (in India and Australia), this could not be calculated as there was no recommendation of a dosage. The largest range was observed in India, where the minimum CMDD was priced at € 0.06 and the maximum CMDD at € 3.52. The mean CMDD was cheapest in the USA at € 0.50 and the most expensive in India at € 0.77. In India and Australia, the median was highest at € 0.64, whereas the lowest median was in the USA at € 0.5. The USA also had the lowest maximum value for CMDD at € 1.2. Germany offered the second-lowest maximum CMDD at € 1.43. The UK observed the second highest range, where the cheapest CMDD was € 0.09 and the most expensive € 3.06; the mean was also the second most expensive, at € 0.63. The data is summarized in Table 5.

**Table 5:** Summary of statistical values for CMDD

	**Mean (€)**	**Median (€)**	**Minimum value (€)**	**Maximum value (€)**
Australia	0.7	0.64	0.13	2.13
Germany	0.6	0.45	0.17	1.43
India	0.77	0.64	0.06	3.52
UK	0.63	0.42	0.09	3.06
USA	0.5	0.5	0.1	1.2

### Comparison of advertising benefits

**Fig. 10:**
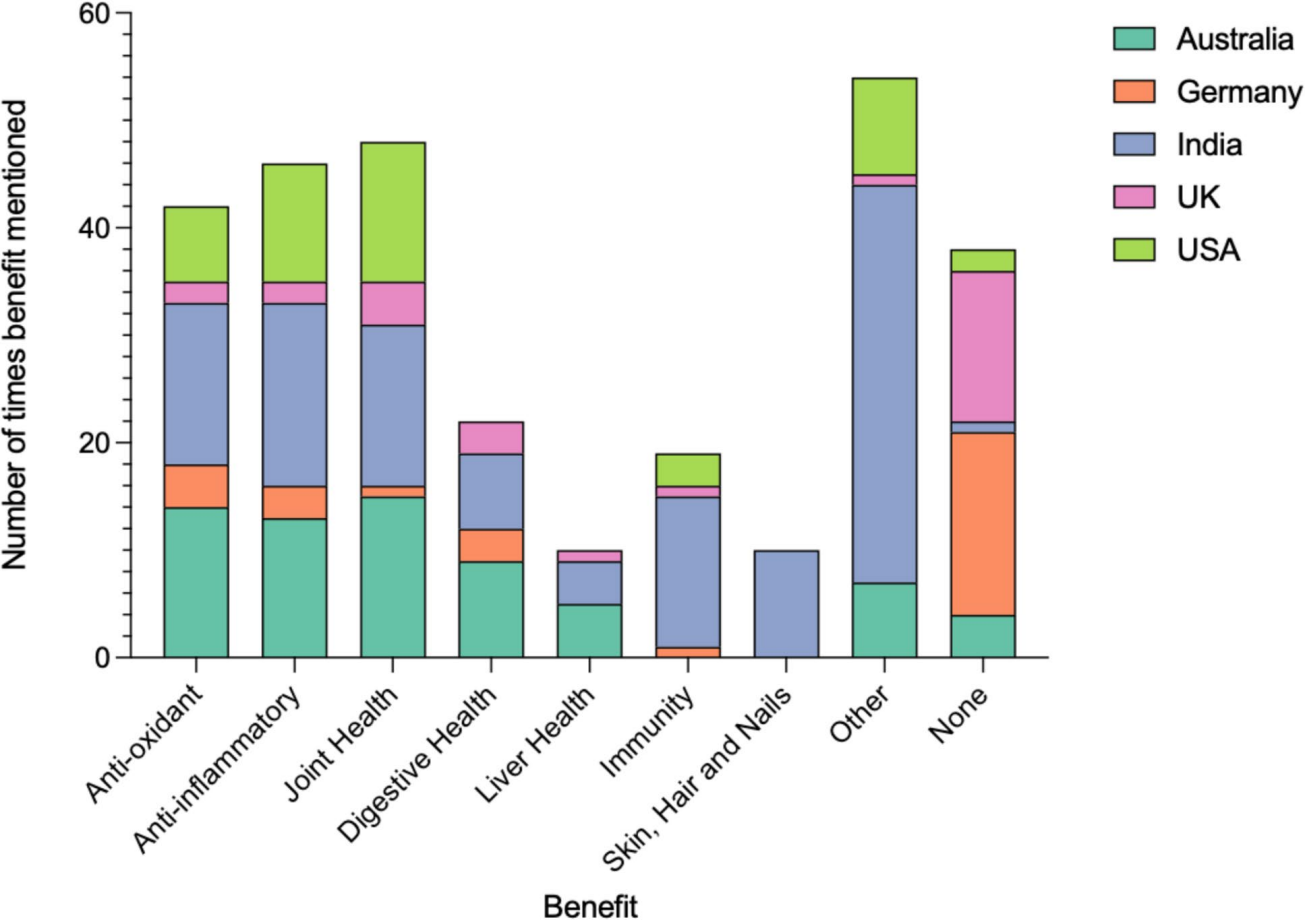
Comparison of advertising benefits. Graphical representation of the number of times a benefit was mentioned in the preparations. The information is presented in a stacked bar chart with each bar showing the number of times a benefit was mentioned per category. The bar is then divided into sections showing the number of times a benefit was mentioned in each country. Each country is represented by a color shown in the top right of the diagram

Figure 10 illustrates the frequency of benefits mentioned on product packaging across the 5 countries. India emerged as the most consistent advertiser, featuring benefits a total of 119 times, with only one supplement omitting any mention. Notably, India also showcased a diverse range of benefits. Australia mentioned benefits 63 times, with 4 preparations omitting any mention. Joint health stood out as the most prevalent benefit in Australia, appearing in 15 out of 25 preparations (60%). Similarly, in the USA, joint health was the most commonly cited benefit, mentioned in 13 out of 25 preparations (52%). Here, benefits were mentioned 43 times. Germany and the UK exhibited a lower frequency of benefits mentioned, 12 and 14 times, respectively. A significant portion of preparations in both countries did not mention any benefits at all (17/25 preparations, 68% for Germany; 14/25 preparations, 56% for the UK).

In the UK, joint health was the most emphasized benefit, appearing in 4 out of 25 preparations (16%). In Germany, antioxidants were the most commonly mentioned benefit, featured in 4 out of 25 preparations (16%). Across all countries, joint health emerged as the most frequently advertised benefit, mentioned a total of 48 times. This was closely followed by mentions of anti-inflammatory benefits (46 times) and antioxidants (42 times).

### Dosage form

**Fig. 11:**
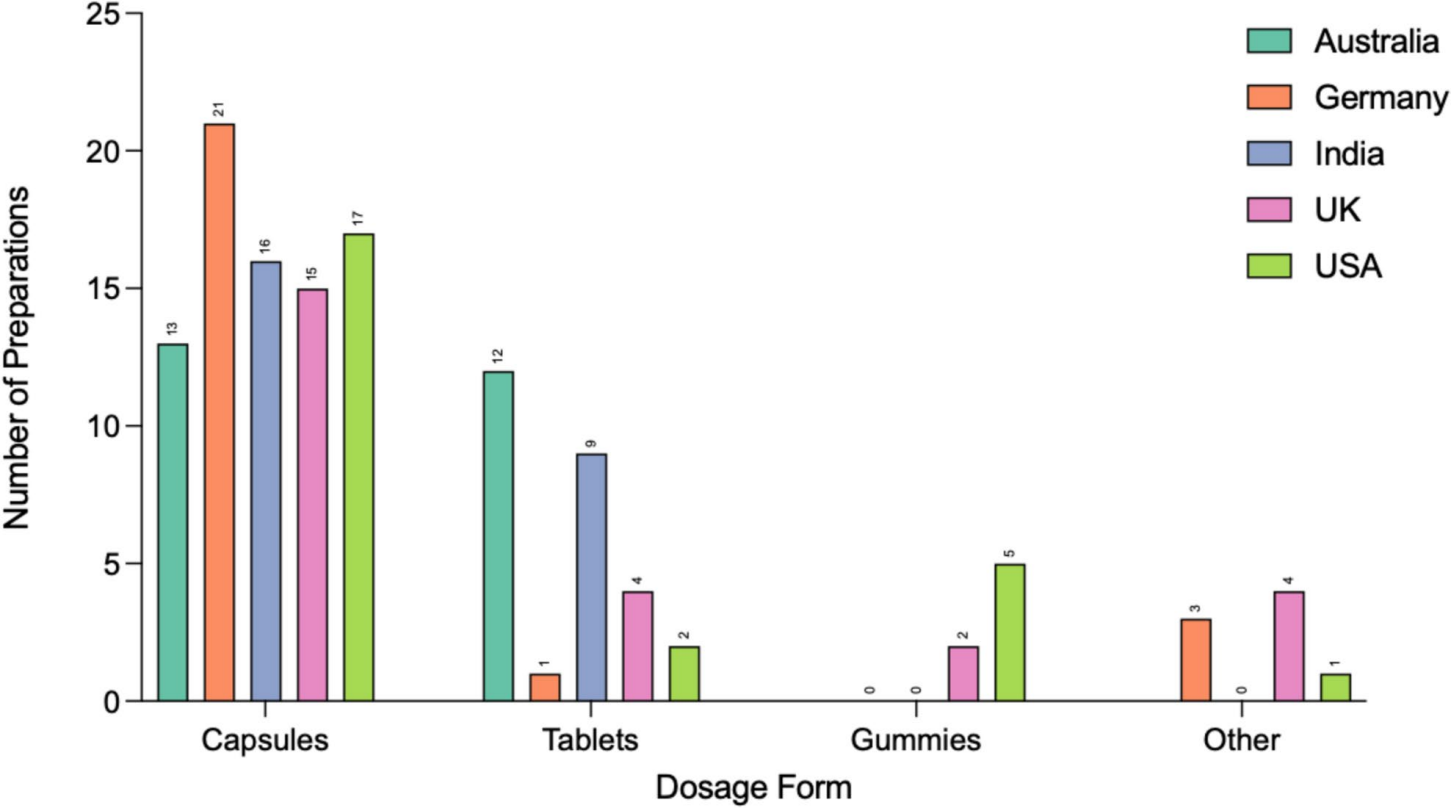
Dosage form. Graphical representation of dosage forms of preparations. Information presented in a grouped column chart

Figure 11 shows the dosage forms for all of the preparations. Capsules were the most common for all countries. In descending order: Germany 21/25 preparations (84%), USA 17/25 preparations (68%), India 16/25 preparations (64%), UK 15/25 preparations (60%), and Australia 13/25 preparations (52%). Australia had a similar number of tablets in 12/25 preparations (48%), whereas Germany offered the least amount of preparations with tablets (1/25 preparations, 4%). The USA offered 5/25 preparations (20%) with gummies and 1 in the “other” category as a “liquid soft-gel.” The UK also offered 2/25 preparations (8%) in gummy form and 4/25 preparations (16%) in “other” forms, including an oral spray and effervescent tablet. Germany offered 3/25 preparations (12%) in “other” forms, including as a liquid.

### Recommended intake

Figure S5 shows the number of preparations which offered a recommendation of intake on the packaging or advertising website. In total, 38/125 preparations (30.4%) offered no recommendation and 75/125 preparations (60%) suggested with meals and/or fluids. The UK and Germany performed best, with 3/25 preparations (12%) displaying no recommendation of intake. In the USA, 8/25 preparations (32%) did not display a recommendation. India and Australia both had 13/25 preparations (52%) with no recommendation.

### Warning of overdose

Figure S6 provides an overview of the number of preparations proving an overdose warning, specifically “do not exceed stated dose,” either on the packaging or advertising website. In Australia, no preparations displayed an overdose warning. In India, 23/25 preparations (92%) did not display a warning. In the USA, 21/25 preparations (84%) did not carry an overdose warning; the MDD of these preparations ranged from 47.5 to 950 mg. A warning was carried on the product offering the highest amount of curcuminoids in the USA, which had an MDD of 2850 mg. Germany had 4/25 preparations (16%) not displaying an overdose warning, where the MDD ranged from 41 to 600 mg, whereas the UK had 3/25 preparations (12%) with no warning, where the MDD was only specified in 1 preparation of 285 mg.

### Indication of adverse effects

Figure S7 shows how many preparations indicated possible adverse effects, either on the packaging or advertising website. No preparations in India indicated any adverse effects. 24/25 preparations (96%) in Australia and the UK did not indicate any adverse effects. One preparation in Australia mentioned the product could affect sugar levels in diabetic patients. One preparation in the UK recommended to consult a doctor if gastrointestinal symptoms occur. 22/25 preparations (88%) in Germany did not indicate any adverse effects. 3/25 preparations (12%) in Germany prohibited the use of supplements in patients with biliary diseases. 18/25 preparations (72%) in the USA did not indicate adverse effects. Five preparations in the USA mentioned to consult a doctor before taking the supplement if the consumer has biliary issues, and 1 preparation also recommended to consult a doctor if a clotting disorder has been diagnosed. One preparation also warned against use if the consumer has excessive stomach acid or ulcers. Two preparations mentioned to consult a doctor if gastrointestinal symptoms occur.

### Indication of possible drug interactions

Figure S8 shows the number of preparations indicating possible drug interactions. 24/25 preparations (96%) in Australia and India did not indicate any possible drug interactions, 23/25 preparations (92%) in Germany and the UK, and 16/25 preparations (64%) in the USA. One preparation in Australia, India, and Germany and 2 preparations in the USA warned against taking the supplement with anticoagulants. One preparation in Germany, 2 preparations in the UK, and 5 preparations in the USA recommended to consult a doctor if taking other medication.

### Warnings for pregnant or lactating women

Figure S9 shows the number of preparations explicitly stating the product is not suitable for pregnant or lactating women on the packaging or on the advertising website. No preparations in India or the USA stated warnings; however, 22/25 preparations (88%) in Australia and Germany and 20/25 preparations (80%) in the UK did. One preparation in the UK stated the preparation was suitable for breastfeeding women, and 1 preparation in Germany stated the preparation was not for pregnant women.

### Naming target audiences

All preparations were analyzed to determine if a target audience was mentioned. This is graphically represented in Fig. S10. 21/25 preparations (84%) in India did not mention a target group, 20/25 preparations (80%) in Germany, 18/25 preparations (72%) in Australia, and 13/25 preparations (52%) in the UK and USA. When a target audience was mentioned in Australia, the USA, and Germany, this was always adults. In the UK, 1 preparation mentioned it was not suitable for childbearing women, 2 were suitable for children over 12, 1 was suitable for children over 3 years old, and 1 specifically mentioned not for children. In India, 1 preparation was deemed suitable for children and 1 warned to consult a doctor before giving to children.

## Discussion

### Analysis of types of turmeric

The FDA is required under the DSHEA (1994) to label dietary supplements as food, making manufacturers subject to GMP guidelines, which include testing for contamination (21 CFR §321(ff)(3)(b)(2). U.S. Food and Drug Administration (2023). However, these guidelines are not binding, and there is no verification of ingredients from the FDA. Manufacturers are not required to confirm the identity of all ingredients supplied to them. Under EU General Food Law Regulation (EC) No 178/2002, food supplements are classed as foodstuffs, where the manufacturer is also responsible for the safety of the product. Similarly, the TGA, FSA, and FSSAI do not verify ingredients or potency of supplements, in contrast to pharmaceuticals. This is concerning considering one study reported that 59% of tested botanical supplements contained plant species not listed on the label (Starr 2015). Although *Curcuma longa* is the most widely cultivated turmeric source, the powder of *Curcuma zedoaria* is a common adulterant in and is known to be toxic (Latif et al. 1979). An analysis in India in 2004 compared 3 turmeric powders with genuine powders of *Curcuma longa* and *Curcuma zedoaria* and discovered a higher presence of *Curcuma zedoaria*, despite the samples meeting the quality standards for curcumin levels (Bejar 2018). Bejar (2018) also reported widespread lead contamination in turmeric, while a more recent large-scale analysis found intentional adulteration, with levels up to 2935 μg/g—over 200 times the FSSAI limit (Forsyth et al. 2024). A newer concern is substitution with synthetic curcumin, which lacks safety evaluation; in one case study of 14 turmeric supplements, only 4 preparations were void of synthetic ingredients (You et al. 2022; Bejar 2018). Germany had the most preparations containing turmeric powder in total (11/25 preparations, 44%), exhibiting the highest prevalence of preparations susceptible to contamination, primarily attributed to the utilization of turmeric sources lacking third-party safety approvals.

### Analysis of turmeric effect enhancing substance

Current research extensively reports the safety of curcumin yet fails to address the potential rise in toxicity concomitant with enhanced curcumin bioavailability through TEES. One study documented an increase in liver injury due to turmeric in the USA and suggested an increased use of black pepper in combination with turmeric as a possible reason (Halegoua-DeMarzio et al. 2023). The BfR reports cases of hepatotoxicity using combination preparations of turmeric with piperine even at doses lower than the ADI (Bundesinstitut für Risikobewertung (BfR), 2021). Nearly half the preparations (58/125, 46.4%) included a TEES, with Germany contributing the most to this statistic by incorporating a TEES in 19/25 preparations (76%). In addition, Germany had the most preparations containing turmeric extract with a TEES (10 preparations of a total of 69 using turmeric extract, 6.9%). Turmeric extract is highly bioavailable and contains 95% curcuminoids, where CUR compromises 70.07%, DMC 20.28%, and BDMC 3.63% (Gouthamchandra et al. 2021). Australia, on the other hand, incorporated a TEES least often (in 8/25 preparations, 32%) and exhibited no complexes with a TEES. Despite this, Australia also most often utilized a complex (7/25 preparations, 28%).

Significantly, out of the 67 preparations that did not include a TEES, 18 preparations were complexes. These formulations increase curcumin’s bioavailability by improving its metabolism, absorption, and permeability. They fall into three main categories: first-generation formulations utilize piperine, turmeric oils, or other natural compounds to inhibit detoxification enzymes, thereby delaying curcumin metabolism and enhancing absorption (Hegde et al. 2023). Second-generation formulations increase solubility of curcumin by using emulsifiers in the form of lipid or phospholipid complexes, among others (Hegde et al. 2023). Here, elevated plasma levels of curcuminoids are predominantly achieved through the formation of conjugated metabolites (Hegde et al. 2023). Third-generation formulations have been shown to have a 100-fold increased bioavailability compared to curcumin by increasing “free” curcuminoids (Hegde et al. 2023). Currently, no health authority approves curcumin as a licensed therapeutic agent; however, complexes are widely used in clinical trials due to their enhanced bioavailability and standardized composition, enabling reliable dosing and therapeutic evaluation. Table 6 summarizes some complexes which have also been used in clinical trials. In total, 8 of the total 26 preparations using a complex also included a TEES, making these extremely highly available preparations (30.7%). This was most pronounced in the USA and Germany (3/26 preparations, 11.5%).

**Table 6:** Clinical applications of curcumin complexes

**Complex**	**Contents**	**Curcuminoids**	**Claimed bioavailability improvement**	**Clinical trial example**	**Generation based on **Hegde et al. (2023)
Meriva®	Curcumin-phosphatidylcholine phytosome	18–22%	30 × (Indena 2019)	( Panahi et al., 2016 ) 87 NAFLD patients, 1000 mg/d for 8 weeks. Serum lipids and uric acid were reduced ( Sunagawa et al. 2015 ) ( Belcaro et al. 2010 ) Table 11	2nd
Theracurmin®	Submicron colloidal dispersion of curcumin	30%	42 × (Theravalues Corporation, n.d.)	( Nakagawa et al. 2014a, b ) ( Sunagawa et al. 2015 ) ( Kanai et al. 2012 ) Table 11	2nd
Longvida®	Solid lipid curcumin particle (SLCP)	20%	> 100 × ( Jamwal et al. 2018 )	( Cox et al. 2015 ) 60 patients, 400 mg/d for 4 weeks, significant improvement in cognition	3rd
BCM-95®/Curcugreen®	Curcuminoids with turmeric essential oil	95%	6.9 × ( Antony et al. 2008 ) )	( Shep et al. 2020 ) 140 OA patients, 500 mg for 28 days, effective in improving pain in combination with NSARs (Sunagawa et al.,^2015^) Table 11	1st
CurQfen®	Curcumin-galactomannoside complex using fenugreek galactomannan (soluble fenugreek fiber)	40%	270 × (Akay Group n.d.)	( Pancholi et al. 2021 ) 20 patients, 500 mg/2xd for 90 days, determined safe for regular consumption	3rd
NovaSol®	Micellar curcumin	95%	185 × ( Schiborr et al. 2014 )	( Schiborr et al. 2014 ) 13 patients, single 500 mg dose, micellar formulation significantly improved bioavailability with observable sex differences	2nd
CurcuWin®	Curcumin + cellulosic and natural polymer carriers (water dispersible UltraSOL technology)	20%	46 ×	( Lopresti et al. 2014 ) ( Thota et al. 2018 ) Table 11	2nd
C3 Complex®	Standardized curcuminoid mixture	95%	None	( Lao et al. 2006 ) Table 11	Point of reference

### Analysis of specified curcuminoid amount per unit (mg)

In total, 43/125 preparations (34.4%) did not specify the curcuminoid amount. These preparations meet the basic requirement of labeling the amount of turmeric but lack information on the active substance content, posing a significant labeling deficiency. Indian preparations lacked this information most often (12/25 preparations, 48%). While Germany showed the best compliance, 2/25 preparations (8%) still failed to state this detail. 9/25 preparations (36%) from Germany had an SCA above 201 mg. Australia and Germany also contained preparations with the highest amount of curcuminoids per unit (2 and 1, respectively) between 501 and 1000 mg, up to more than triple the recommended ADI in one unit. Although Germany’s high transparency in advertising, this ultimately led to them having the most preparations with a high SCA per unit.

A single preparation contained less than 10 mg of curcuminoids per unit; the preparation was from the UK. This raises questions about the minimum dosage for a therapeutic effect. Economic interests of manufacturers possibly play a role here, as a lower SCA per unit would result in more units per day and therefore more product to achieve a substantial effect.

### Analysis of maximum daily dose (MDD) (mg)

A potential issue for consumers arises from the fact that in 44/125 preparations (35.2%), the MDD could not be calculated as the amount of active substance or recommended units per day is not specified. Not only does this risk overdose, but this may be relevant to consumers who desire a certain benefit. German preparations most often cited an MDD (22/25 preparations, 88%), whereas India stated an MDD least often (13/25 preparations, 52%). The following data must also be considered with the fact that 9 preparations in Australia, 3 in Germany, 12 in India, 10 in the UK, and 10 preparations in the USA did not state an MDD.

The UK stands out with a lower mean dose (123.4 mg) compared to other countries, and its median dose (110 mg) is also relatively low. Although the UK is the only country with an average dose less than the ADI for an average adult, this dosage is almost half that of the JEFCA recommendation, raising concerns of an effective dose. The maximum value in supplements in the UK was 475 mg, which is significantly lower than the other countries. Australia and India exhibit higher average dosages, with mean values of 422.2 mg and 457.4 mg, more than double that of the recommended ADI. However, the median of 240 mg in Australia is significantly lower than that of India (380 mg), suggesting the tendency for higher curcumin concentrations is more prevalent in India.

An Acceptable Daily Intake (ADI) for curcumin was established in 2004 by JEFCA, recommending a dosage of 0–3 mg/kg of body weight based on a NOEL of 250–320 mg/kg of body weight with a safety factor of 100 (JECFA 2004). This recommendation was derived from a multigenerational reproductive toxicity study done in Wistar rats (Ganiger et al. 2007) and encompasses the total daily curcumin intake, including that from both dietary sources and supplements, without a TEES. The FSSAI, FDA, and TGA do not officially recommend a dosage, relying on the manufacturers to recommend an appropriate dosage based on current scientific evidence. However, the JEFCA recommendation is commonly referenced internationally as a general guideline. For an average adult weighing 70 kg, the recommended daily curcumin intake, according to the WHO, would be 210 mg. Using the gathered data, 36/125 preparations (28.8%) were discovered to recommend a dosage surpassing the ADI. In absolute terms, this was most often the case in Germany, where 11/25 preparations (44%) recommended an MDD above the ADI. Of these preparations, 10 utilized a TEES. This means not only were these preparations above the recommended ADI, but they were also highly available preparations which have not been assessed for safety. The UK had the most preparations recommending a dosage less than the ADI (12/25 preparations, 48%) as well as the least preparations recommending a dosage higher than the ADI (3/25 preparations, 12%). Relatively, India had the most preparations above the ADI (8/13 preparations, 61.5%).

### Analysis of cost of maximum daily dose (CMDD) (€)

According to a market analysis report, the global curcumin market size was valued at $ 58.2 million in 2020 and is expected to grow at a CAGR of 16.1% from 2020 to 2028. Pricing is generally not regulated by government authorities and is primarily influenced by marketing dynamics. American supplements had the lowest mean (€ 0.50) and maximum (€ 1.20) prices, with a symmetrical price distribution suggesting good pricing regulation. India as a major producer of turmeric showed the widest price range, with the highest maximum (€ 3.52) and mean (€ 0.77) prices and the lowest minimum (€ 0.06), indicating a less regulated market (Prasad et al., 2011). The UK and Germany had the lowest medians (€ 0.42 and € 0.45) but higher means (€ 0.63 and € 0.60). Thus, American supplements are best regulated in terms of pricing, while Indian supplements appear the least regulated.

Price can be indicative of quality, with consumers associating higher prices with superior product quality. Higher prices may also be justified by additional features, such as enhanced bioavailability or specific formulations. For example, a preparation in the UK costing > € 2.0 for 100 mg of curcuminoids is administered as a buccal spray, yet each dose offers a mere 6.75 mg curcuminoids. Marketing and production costs likely influence pricing; however, a correlation between curcuminoid content and price remains unclear due to 35.2% of preparations failing to mention the concentration of curcuminoids.

### Comparison of marketing benefits

Health claims on supplements in the EU require EFSA approval under Regulation (EC) No 1924/2006, which was retained by the UK in the Nutrition and Health Claims Regulation (Reg. EU 1924/2006; NHC Reg. EC/1924/2006). Currently, the one registered claim: “Curcumin contributes to the normal functioning of joints” was rejected due to insufficient evidence after EFSA analyzed 16 studies investigating curcumin’s role in osteoarthritis and rheumatoid arthritis (Turck et al. 2017  Commission Regulation (EU) 2018/1556; EU Register of Health Claims, n.d; Nutrition and Health Claims Register (NHC), n.d). Therefore, related claims on 4 UK and 1 German preparation were incompliant. No other authorized claims exist. Antioxidant claims, seen in 4/25 (16%) of German products, may fall under Article 13(5) but still require EFSA approval before use. The remaining benefit claims—11 in Germany (32%) and 10 in the UK (44%)—were not EFSA-evaluated and therefore not authorized as health claims.

In the USA, claims regarding product benefits are permissible only with substantiated evidence ensuring they are truthful and not misleading (21 CFR §101.93. U.S. Food and Drug Administration (2023). Since 2019, 30 curcumin preparations have received warning letters due to false anti-disease claims (Warning Letters: Curcumin, n.d). For example, curcumin is an antioxidant and an anti-inflammatory and improves joint health. The phrasing is crucial and plays a decisive role, as function claims for maintenance and support are permitted. Notification to the FDA within 30 days of product launch is mandatory, alongside inclusion of a prescribed disclaimer. This leaves a window for customers to purchase products with misleading claims and leaves the responsibility with the consumer to assess the current research independently, leaving vulnerable demographics at risk.

The FSSAI allows statements relating to the structure or function or the general well-being of the body if the statement is supported by the generally accepted scientific data (Food Safety and Standards Regulations (FSSR) (2016)). There is no third-party evaluation of these statements, leaving this regulation ambiguous.

As a result, benefits were marketed significantly more often here (119 times) in comparison to other countries and were also the most diverse.

The TGA lists curcumin as a complementary medicine which allows these supplements to carry “low-level” indications from a list of permitted indications, if backed by either scientific or traditional evidence (Permissible Indications Determination, 2021). However, this requirement is not regulated before marketing, and no disclaimer is attached. Therefore, the 63 times benefits were mentioned did not undergo pre-market evaluation as to whether enough scientific evidence supports them.

Across all countries, joint health emerged as the most frequently advertised benefit, mentioned a total of 48 times; however, only in the UK and Germany was this claim analyzed for its accuracy. Particularly concerning are the 5, 4, and 1 times liver health was mentioned as a benefit in Australia, India, and the UK, respectively, when numerous reports have suggested a link between turmeric supplementation and hepatotoxicity (Domenico Gallina et al. 2024; Luber et al. 2019; Lee et al. 2020; Lukefahr et al. 2018).

### Dosage form

The USA had 5/25 preparations (20%) in the form of gummies. Notably, 1 of these gummies contained an MDD of 500 mg, exceeding twice the ADI recommended by JEFCA for the average adult (JEFCA, 2004). Two of these preparations also did not mention an MDD. The UK offered 2/25 preparations (8%) as gummies, both of which did not state an MDD. Gummies may present a higher risk of overdose due to their resemblance to candy, potentially leading to accidental ingestion by children. This is especially concerning if the gummies do not clearly indicate the MDD, as individuals may unknowingly consume excessive amounts, increasing the risk of overdose.

The UK offered 4/25 preparations (16%) in “other” forms, including an oral spray and effervescent tablet. Germany offered 3/25 preparations (12%) in “other” forms, including as a liquid. These dosage forms may change the pharmacokinetic profile and therefore the bioavailability, as well as offering a variable SCA per unit.

### Recommended intake

In the EU and the UK, Annex I of Directive 2002/46/EC does not explicitly state “directions for use” but requires that the label provides clear and accurate information on any specific warnings or precautions necessary for the safe use of the supplement (Dir 2002/46/EC). The UK and Germany both had 3/25 preparations (12%) with no recommendation of intake. Similarly, in the USA, the requirement for directions for use on dietary supplement labels in the USA is inferred from broader labeling provisions outlined in 21 CFR 101.36; historically, enforcement actions have contributed to the expectation for directions for use to be stated on health supplements. 8/25 preparations (32%) in the USA did not display a recommendation. Under the Therapeutic Goods Regulations 1990 and FSSR, Australia and India demonstrated the same approach but had significantly more preparations lacking a recommendation (13/25 preparations, 52%).

Notably, since curcumin is fat-soluble, its bioavailability is enhanced through improved absorption when consumed with a high-fat meal or drink (Stohs et al.  2020). On the other hand, it is not known if the gastrointestinal symptoms mentioned in 4.10 may exacerbate if taken on an empty stomach. These aspects must be taken into consideration by manufacturers when recommending a method of intake. Furthermore, the majority of dosage forms are in capsule and tablet form which can present as a choking hazard and should therefore be recommended to be taken with fluids.

### Warning of overdose

The safety of curcumin has not been thoroughly investigated. One study suggested the intake of curcumin over a long period of time could lead to hepatotoxicity due to an increase, and therefore imbalance, in reactive oxidative species and pro-inflammatory cytokines (Qiu et al. 2016). Another reported cephalgia, yellow stools, rashes, and diarrhea at doses over 12 g/day (Bandyopadhyay, 2014). Given the unrestricted availability of these preparations, achieving such a dose over an extended period is feasible.

Under European Directive 2002/46/EC Art. 6(2c) and the Food Supplements Regulations (FSR) 2003 (No. 1387, No. 278, No. 1719, and No. 273), dietary supplements in Germany and the UK, respectively, are required to carry a warning not to exceed the stated recommended daily dose (Dir 2002/46/EC). The UK most often stated a warning (22/25, 88%), followed by Germany (21/25, 84%). However, although these preparations labeled the amount of turmeric and recommended a number of units to intake per day, superficially complying with Art. 6(2b) which states a portion of the product must be recommended for daily consumption, the lack of information pertaining to the amount of active substance, paired with the absence of an overdose warning, raises safety concerns.

There is no requirement to state an overdose warning in the USA (21 CFR §101.17. U.S. Food and Drug Administration (2023). Curcumin received a no objection letter in 2019 to be generally recognized as safe (GRAS) by the FDA (GRN No. 822, n.d). However, the toxicology evaluation did not evaluate the safety of preparations with enhanced bioavailability. Similarly in Australia, under TGO No. 92, this is not mandatory. On the other hand, health supplements in India are required to state the warning “not to exceed the recommended daily usage” (Food Safety and Standards Regulations (FSSR) (2016)). As 24/25 preparations (96%) did not state this, enforcement of this regulation appears to be severely lacking.

### Indication of adverse effects

Gastrointestinal symptoms such as diarrhea, rash, headache, and yellow stools were reported in 7 subjects receiving between 500 and 12,000 mg within 72 h (Hewlings et al. 2017). Another study where participants ingested 0.45 to 3.6 g/day of curcumin for 1 to 4 months reported nausea and diarrhea, along with elevated levels of serum alkaline phosphatase and lactate dehydrogenase (Hewlings et al. 2017). These adverse effects occurred without the addition of a TEES, which is often the case in studies concluding curcumin’s safety. As of June 2023, the TGA received 18 reports of hepatotoxicity linked to *Curcuma longa* (turmeric) and/or curcumin products (TGA 2023a, b). Nine cases indicated a potential connection to the products, with 4 cases showing no other likely contributing ingredients. Among these, 2 cases were severe, including 1 resulting in death. France’s nutrivigilance program recorded more than 100 adverse event reports, including 15 cases of hepatitis, potentially linked to the use of dietary supplements containing turmeric or curcumin, which led the agency to advise people with bile duct diseases against consumption of these supplements (ANSES 2022). Doses as low as 20 mg demonstrated a positive cholekinetic effect; therefore, unregulated dosage recommendations pose a potentially fatal outcome (Rasyid et al. 2002). The USA most often mentioned the possibility of adverse effects (7/25 preparations, 28%), and specifically 5 preparations mentioned that these supplements are not recommended for patients with gallstones. Two preparations mentioned possible gastrointestinal discomfort. Germany had 3/25 preparations (12%) which did not recommend this product for people with gallstones. The UK mentioned gastrointestinal discomfort in 1 preparation. The Australian and Indian supplements did not state either of these adverse effects. In conclusion, the findings indicate that American dietary supplements were the most proactive in advising against potential adverse effects and also demonstrated the most up-to-date information regarding adverse effects.

### Indication of possible drug interactions

A review of pharmacokinetic interactions with conventional medications showed curcumin can alter the peak serum concentration when concomitantly administered with cardiovascular drugs, antidepressants, anticoagulants, antibiotics, chemotherapeutic agents, and antihistamines, although only one study proved a significant change (Bahramsoltani et al. 2017). Only 1 preparation in Australia warned against taking the preparation with a diagnosed clotting disorder. One preparation in Germany and the USA warned to not take the supplements with anticoagulants, whereas 1 preparation in India warned to discontinue the supplements 2 weeks before a surgery. Numerous studies have commented on the anticoagulatory properties of curcumin, stating the aPTT and PT were significantly prolonged and suggesting that curcumin should even be considered as an adjunctive to current anticoagulant and antiplatelet therapy (Kim et al. 2012; Keihanian et al. 2017). The data reveal a significant shortcoming in the labeling of curcumin supplements. The USA had a significantly higher number of preparations (9/25 preparations, 36%) recommending consulting a doctor if taking medication, demonstrating again better regulation of supplements. Table 7 illustrates the possible drug interactions of common medications in further detail.

**Table 7:** Pharmacological interactions of curcumin with common medications

Drug class	Examples	Effect of curcumin	Clinical implications	Notes	Evidence
Anticoagulants/antiplatelets	Warfarin, acetyl salicylic acid, clopidogrel	Inhibits platelet aggregation via suppression of thromboxane A2 synthesis and COX-1/COX-2. Inhibits vitamin K epoxide reductase (VKOR)	Increased risk of bleeding and bruising. Monitor PT/INR levels in warfarin users. Patients should be counseled on signs of excessive bleeding	Higher risk in patients with comorbidities, e.g., liver cirrhosis	Clinical trial in mice ( Kim et al., 2012 )
Cyclooxygenase (COX) inhibitors	Ibuprofen, diclofenac	Inhibits COX-2 and reduces prostaglandin E2 (PGE2) synthesis. Acts on nuclear factor-kappa NF-κB	May provide synergistic anti-inflammatory effects and potentially reduce the required COX inhibitor dose	Mitigate COX inhibitor-induced gastrointestinal damage	Randomized trial of 140 patients with knee osteoarthritis receiving 1 g curcumin complex with 100 mg diclofenac for 4 weeks (Shep et al. 2020)
Chemotherapeutic agents	Doxorubicin, cisplatin, paclitaxel	Inhibits NF-κB and STAT3 pathways. Inhibits P-glycoprotein, reducing drug efflux, and enhancing drug retention in cancer cells	Enhances the efficacy of chemotherapy, particularly in resistant tumors. Potential for reduced toxicity by mitigating oxidative damage	Should only be used under supervision in oncology settings. May reduce cardiotoxicity associated with doxorubicin	In vivo and in vitro studies ( Tan et al. 2019 )
Antihypertensive drugs	Lisinopril, captopril	Inhibits NF-κB and activates endothelial nitric oxide synthase (eNOS), leading to vasodilation. Inhibits UGT and P-gp	Higher plasma concentrations and slower breakdown of drugs, increasing risk of adverse effects. Increase risk of hypotension	Monitor blood pressure regularly	Data-mining study ( Prieto-Garcia et al. 2023 )
Antidiabetic drugs	Metformin, sulfonylureas	Enhances insulin sensitivity via activation of AMPK and suppression of oxidative stress. Inhibits TNF-α and IL-6, reducing insulin resistance	Risk of hypoglycemia when combined with antidiabetic drugs. Potential for reduced medication dose requirements	Monitor blood glucose regularly. Risk may be higher in patients with renal impairment	Systematic review and meta-analysis, treatment > 8 weeks and dose > 300 mg/d curcuminoids ( Yuan et al. 2019 )
Antibacterial drugs	Ciprofloxacin, tetracyclines	Inhibits bacterial biofilm formation through quorum sensing inhibition and modulates ROS generation	May improve efficacy against resistant bacterial strains	Monitor for potential synergy in cases of multidrug-resistant infections	In vitro studies ( Tyagi et al. 2015 )
Immunosuppressant drugs	Cyclosporine, tacrolimus	Inhibits IL-2 production and T cell proliferation via suppression of NF-κB	Risk of reduced efficacy of immunosuppressants in transplant or autoimmune patients	Modulates immune function in diseases requiring strict immune suppression, e.g., psoriasis	In vitro studies ( Bharti et al. 2010 )
Antidepressant drugs	Fluoxetine, sertraline	Increases serotonin and dopamine levels by inhibiting monoamine oxidase (MAO-A). Modulates brain-derived neurotrophic factor (BDNF) expression	May improve outcomes in depression, particularly treatment-resistant cases	Monitor for serotonin syndrome when used with other serotonergic agents	In vitro study on rats ( Khatri et al., 2016 ), meta-analysis with 200 to 1820 mg/d of curcumin for 8 to 12 weeks ( Sarraf et al., 2019 )
Antiepileptic drugs	Phenytoin, valproic acid	Reduces neuronal excitability through modulation of calcium and sodium channels and inhibition of oxidative stress pathways	Potential for additive seizure control but may affect hepatic metabolism of antiepileptics via cytochrome P450 (CYP) enzymes		In vivo study in rats ( Reeta et al. 2011 )
HMG-CoA reductase inhibitors	Atorvastatin, simvastatin	Inhibits HMG-CoA reductase activity and reduces LDL oxidation. Suppresses NF-κB and reduces vascular inflammation	Additive lipid-lowering effects. Increased risk of statin-induced myopathy or liver enzyme elevation	Monitor liver function tests (LFTs) and muscle symptoms	Data-mining study ( Prieto-Garcia et al. 2023 )

### Warnings for pregnant or lactating women

Studies investigating curcumin in pregnant or lactating women are limited and conflicting. Although in vitro and in vivo studies have suggested a beneficial role in common pregnancy complications, its role in early pregnancy has not been established (Tossetta et al 2021). Furthermore, as mentioned previously, supplement ingredients are not regulated, meaning their safety and potency are not guaranteed. Given the vulnerability of this demographic, a warning to inform pregnant women to refrain from taking the supplements should be stated until rigorous studies conclude their safety. Despite a significant portion of preparations still lacking warnings, UK preparations exhibited safer labeling practices by stating a warning most often (5/25 preparations, 20%), whereas no warnings were provided on American or Indian preparations.

### Naming target audience

Targeting based on the JEFCA recommendation would ensure safer supplements; however, 2 preparations in India mentioned children as a target audience—one for ages 5–12—without mention of an MDD. In the UK, 3 supplements were labeled suitable for children (2 preparations for children over 12, 1 for children over 3 years old), and 4 explicitly stated they were not recommended for children, irrespective of the MDD. Dosage form appeared to play a role in the UK, as the oral spray was recommended for children over 3 years; however, there was no observable relationship between gummy forms and recommendations for children. The USA, Australia, and Germany predominantly target adults, reflecting caution, as the safety of curcumin in the pediatric population remains unestablished. Overall, regulation of target audiences appeared inconsistent, with the USA showing the most proactive approach in safeguarding vulnerable populations through adult-focused targeting in 12/25 preparations (48%).

### Country comparison

Table 8 summarizes the country comparisons. Germany had the highest number of products susceptible to contamination (44%), while the USA had the fewest (20%). German supplements also most often added a TEES (76%), whereas Australia the least (32%). In terms of bioavailable preparations, the USA performed worst, followed by Germany, whereas Australia performed best. However, this assessment does not consider that complexes typically have higher bioavailability than turmeric extracts, thus not reflecting the true extent of bioavailability differences among the products. This means that although the USA and Germany might have more bioavailable preparations overall, the assessment does not adequately differentiate between the levels of bioavailability provided by different types of preparations. Germany was the most transparent in offering information regarding the SCA (8% not mentioned); however, they also had the highest number of preparations with an SCA above 201 mg (36%). Relatively, India had the most preparations offering an SCA above 201 mg (6/13 preparations, 46.1%) and was also the least transparent in offering this information (48% not mentioned). The UK had the least number of preparations offering an SCA above 201 mg (12%). Germany was again the most transparent in offering information about the MDD (12% not mentioned); however, this showed them to be the least compliant with the ADI absolutely (44% > ADI). India was the least transparent in discussing MDD (52%) and also exhibited the highest mean (547.4 mg). India also had relatively more preparations above the ADI (61.5%). The UK performed most favorably, showing the lowest dosages and best compliance to the ADI (12% > ADI). The CMDD was on average cheapest in the USA (€ 0.5) and highest in India (€ 0.77). Marketing benefits were significantly increased in India and best regulated in Germany. Australia and India least often gave a recommended intake (52%), as opposed to Germany and the UK (12%). Overdose warnings were also most often stated in the UK (12% no warning) and least in Australia (100% no warning), where it is also not mandatory. Adverse effects were best labeled in the USA (79% no indication) and least in India (100% no indication). This was also the same for indicating drug interactions, with Australia tying with India in last place (64% no indication in the USA, 96% no indication in Australia and India). Warnings against pregnant or lactating women taking the supplements were best documented in the UK (80% no warning) and least in the USA and India (100% no warning). Consideration of target audience was best exhibited in the USA (52%) and worst in India (84%). Overall products in the UK were ranked most favorable (37/90), followed by the USA (42/90), Australia (46/90), Germany (49/90), and India (68/90).

**Table 8:** Country comparison

Parameter	Criteria	Australia	Germany	India	UK	USA
Types of turmeric	Preparations using turmeric powder	2 (24%)	5 (44%)	2 (24%)	2 (24%)	1 (20%)
Turmeric effect enhancing substance	Preparations adding a TEES	1 (32%)	5 (76%)	3 (30%)	4 (44%)	2 (36%)
Number of highly bioavailable preparations	i. Turmeric extract ii. Turmeric extract + TEES iii. > 1 Turmeric type + TEES iv. Complex v. Complex + TEES	i. 1 (32%) ii. 1 (5.8) iii. 1 (0%) iv. 5 (28%) v. 1 (0%)	i. 3 (60%) ii. 5 (14.5%) iii. 3 (15.6%) iv. 2 (16%) v. 5 (11.5%)	i. 5 (68%) ii. 3 (8.7%) iii. 3 (15.6%) iv. 1 (12%) v. 2 (3.8%)	i. 2 (56%) ii. 3 (8.7%) iii. 2 (6.3%) iv. 3 (20%) v. 2 (3.8%)	i. 3 (60%) ii. 2 (7.2%) iii. 5 (21.9%) iv. 3 (20%) v. 5 (11.5%)
Specified curcuminoid amount per unit	Relative percentage of preparations > 201 mg only from preparations offering required information	1 (31.3%)	3 (39%)	5 (46.1%)	4 (40%)	2 (33%)
Maximum daily dose	Relative percentage of preparations where MDD > ADI only from preparations offerings required information	3 (50%)	3 (50%)	5 (61.5%)	1 (20%)	2 (35%)
Cost of maximum daily dose	Mean (€)	4 (0.7)	2 (0.6)	5 (0.77)	3 (0.63)	1 (0.5)
Marketing benefits	Number of times benefit is mentioned	4 (63)	1 (12)	5 (119)	2 (14)	3 (43)
Recommended intake	Preparations offering no recommendation	5 (52%)	1 (12%)	5 (52%)	1 (12%)	2 (32%)
Warning of overdose	Preparations without warnings	5 (100%)	2 (16%)	4 (92%)	1 (12%)	3 (84%)
Indication of adverse effects	Preparations with no indication	3 (96%)	2 (88%)	5 (100%)	3 (96%)	1 (79%)
Indication of drug interactions	Preparations with no indication	5 (96%)	2 (92%)	5 (96%)	2 (92%)	1 (64%)
Warnings for pregnant or lactating women	Preparations without warnings	2 (88%)	2 (88%)	5 (100%)	1 (80%)	5 (100%)
Naming target audience	Preparations not naming a target audience	2 (72%)	3 (80%)	5 (84%)	1 (52%)	1 (52%)
Total/90		46	49	68	37	42

Comparison of the analyzed parameters of turmeric supplements in Australia, Germany, India, UK, and the USA using a ranking scale from 1 to 5, with 1 representing the most favorable outcome and 5 representing the least favorable outcome as perceived by the authors.

Table 9 summarizes the regulatory landscape of curcumin supplements internationally but can be extrapolated to apply broadly to all dietary supplements. It highlights key regulatory gaps, including the absence of mandatory ingredient verification, inconsistent labeling requirements, and limited pre-market safety oversight.

**Table 9:** Selected overview of national regulatory references for curcumin supplements

Parameter	USA	EU/UK	India	Australia	Germany
Legal classification	Food (DSHEA 1994 ); supplements regulated as food (21 CFR §321(ff)(3)(b)(2). U.S. Food and Drug Administration, ( 21 2023 )	Foodstuff; manufacturer responsible (Reg. (EC) 178/2002; Dir 2002/46/EC)	Traditional food (FSSR 2011)	Complementary medicine (TGO No. 92, n.d)	Foodstuff; post-market control (Dir 2002/46/EC)
MDD and dosage	Label required: 21 CFR §101.36. U.S. Food and Drug Administration 2023; no official limit; JECFA ADI (2004)	Label required: Dir 2002/46/EC; JECFA ADI (2004)	Not always labeled; JECFA ADI; FSSR 2011	No official limit; JECFA ADI; TGO No. 92	Label required: Dir 2002/46/EC; JECFA ADI (2004)
Marketing claims	Allowed with disclaimer: 21 CFR §101.93. U.S. Food and Drug Administration (2023)	Must be EFSA-approved (Reg. (EU) 1924/2006); retained in UK	Allowed if supported (FSSR 6(3)(ii), 2016)	Low-level claims allowed ( Permissible Indications Determination, 2021 )	Reg. (EU) 1924/2006; EFSA approval required
Overdose warnings	Not required: 21 CFR §101.17. U.S. Food and Drug Administration (2023)	Mandatory: Dir 2002/46/EC Art. 6(2c); FSR 2003 in UK	Required by law: Food Safety and Standards Regulations (FSSR) (2016)	Not mandatory (TGO No. 92, n.d)	Mandatory: Dir 2002/46/EC Art. 6(2c); FSA enforcement
Target audience	Must be specified: 21 CFR §101.36. U.S. Food and Drug Administration (2023)	Must be appropriate and safe: Dir 2002/46/EC	Children mentioned without clear rules: FSSR 2011	Not clearly regulated	Must be safe and appropriate: Dir 2002/46/EC

### Turmeric overdose and intoxication

A NOAEL of 250–320 mg/kg body weight was stated by the BfR, stating however that the absence of undesirable effects could not be assumed. There is no defined maximum tolerated dose for curcumin, and the long-term effects of chronic low-dose supplementation have not been extensively studied. Although turmeric is generally assumed to be safe, toxicity research remains limited because of this assumption. The LD50 in rats is around 5000 mg/kg (Aggarwal et al. 2016), suggesting that the overdose threshold is high in humans; however, acute gastrointestinal issues have been observed at lower doses. For example, among others, one study (Lao et al 2006) reported mild gastrointestinal symptoms at doses up to 12 g over a 3-month period, signaling acute toxicity. Chronic supplementation toxicity, however, is more realistic and concerning, with symptoms including liver damage, gastrointestinal disturbances, and increased bleeding risk, especially in patients with predisposing conditions. Table 10 summarizes three cases of intoxication linked to low-dose turmeric supplements (Bethesda 2024).

**Table 10:** Cases of turmeric overdose and intoxication

Patient information	Symptoms	Dosage and duration	Liver test abnormalities	Outcome
57-year-old woman	Fatigue, nausea, abdominal pain, itching, dark urine, jaundice	Turmeric, 500 mg/day for **2–3 weeks**	- Bilirubin: 9.1 mg/dL - ALT: 1425 U/L - AST: 1374 U/L - ALP: 250 U/L	Liver tests normalized within 6 weeks after stopping turmeric, asymptomatic after 1 year
35-year-old man	Fatigue, low-grade fever, nausea, vomiting, itching, dark urine, jaundice	Complex with TEES, 1000 mg/day for **2 months**	- Bilirubin: 12.5 mg/dL - ALT: 2014 U/L - AST: 796 U/L - ALP: 124 U/L	Jaundice persisted for 2 months; liver tests normalized within 2 months after stopping curcumin
62-year-old woman	Fatigue, nausea, dark urine, jaundice	**Turmeric extract,** 500 mg/day for **14 months**	- Bilirubin: initially 2.5 mg/dL, increased to 13.5 mg/dL - ALT: 1230 U/L - AST: 1628 U/L - ALP: 329 U/L	Worsened condition, **liver failure**, listed for transplantation but died from myocardial infarction and multiorgan failure

### Dose–response relationship

**Fig. 12:**
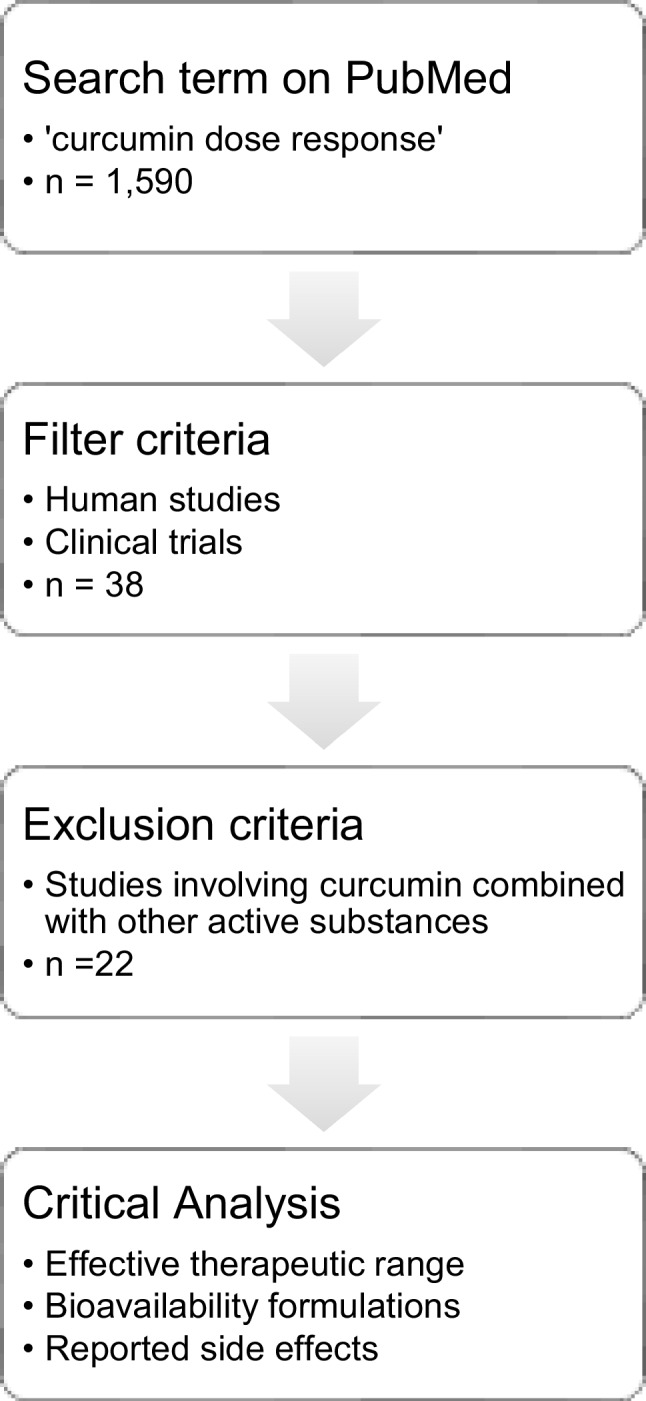
Flowchart showing study review selection and analysis

A focused mini-review was conducted using PubMed. The search term “curcumin dose response” yielded 1590 results. Filters were applied to select human studies and clinical trials, narrowing the pool to 38 studies. To isolate the effects of curcumin alone, studies that involved combination with other active substances were excluded, resulting in 22 eligible studies. These studies were then critically analyzed to assess the effective therapeutic range, the formulations used to enhance bioavailability, and any reported side effects across different dosing regimens. This process is summarized in Fig. 12. The results are reported in Table 11.

Findings from this review indicate that curcumin demonstrates therapeutic efficacy across a relatively broad dose spectrum, with most clinical benefits observed at daily doses ranging from 180 to 2000 mg. For instance, improvements in inflammatory conditions such as osteoarthritis and rheumatoid arthritis were reported at doses as low as 180 mg/day when enhanced bioavailability formulations like Theracurmin or Meriva were used. Similarly, in studies targeting depression and metabolic disorders, standard curcumin at 500–1000 mg/day showed significant clinical benefit. Higher doses, including those above 4000 mg/day and up to 12,000 mg in single-dose escalation studies (Lao et al. 2006), were generally well tolerated, although minimal toxicity was noted in about 30% of participants, and such high doses did not consistently demonstrate proportional increases in efficacy.

Studies using nanoparticle-based or phospholipid-enhanced formulations reported therapeutic effects at markedly lower doses compared to those using unmodified curcumin. This highlights a non-linear dose–response curve where improved absorption can shift the effective therapeutic window, reinforcing the need for formulation-specific dosage recommendations rather than generalized dosing guidelines. It also suggests that higher doses of poorly absorbed curcumin may not be necessary—and in some cases, may be less effective—than lower doses of more bioavailable forms. Understanding and accounting for this non-linear dose response is critical to maximizing curcumin’s clinical utility while avoiding unnecessary exposure or potential adverse effects at high doses.

Importantly, side effects across studies were generally mild, with gastrointestinal discomfort being the most commonly reported at higher doses. Intravenous formulations, such as Lipocurc, showed dose-limiting toxicities at the upper end of the dosing spectrum, reinforcing the importance of formulation and administration route in assessing safety.

While curcumin appears safe across a wide range of doses, its clinical effectiveness is contingent on bioavailability, and higher doses do not necessarily translate into greater benefit. Therefore, establishing optimal dosing parameters based on well-characterized formulations is essential for maximizing therapeutic outcomes while minimizing risks. These findings underscore the need for standardization in curcumin dosing guidelines.

**Table 11:** Studies in review of curcumin dose–response relationship

Study (author, year)	Type	Population	*n*	Dose and duration	Findings	Bioavailability	Side effects	Notes
Amalraj et al. 2017	RCT, double-blind, placebo-controlled	Rheumatoid arthritis patients	36	250 or 500 mg twice daily for 90 days	Significant improvement in symptoms and markers vs. placebo	Natural turmeric matrix (enhanced)	Not mentioned	–
Belcaro et al. 2010	Prospective, controlled study	Osteoarthritis patients	100	Meriva®, 8 months	Significant improvement in pain, joint function, and inflammatory markers vs. control	Meriva®	Not mentioned	Excellent tolerability reported
Chainani-Wu et al., 2011	Randomized, double-blind, placebo-controlled trial	Oral lichen planus patients	20	6000 mg curcuminoids/day (3 divided doses) for 14 days	Significant improvements	Not mentioned	“Uncommon”	No details on side effects
Cheng et al. 2001	Phase I clinical trial	High-risk or pre-malignant lesion patients	25	Oral curcumin: 500–8000 mg/day for 3 months	Not toxic up to 8000 mg/day	Standard curcumin	Not mentioned	–
Golombick et al. 2012	Randomized, double-blind, placebo-controlled cross-over + open-label extension	Monoclonal gammopathy of undetermined significance and smoldering multiple myeloma patients	36 (25 completed 4 g, 18 completed 8 g)	4 g/day for 3 months (cross-over), optional 8 g/day (open-label)	Improved resorption markers and trend toward lower serum creatinine	Standard curcumin	2 patients withdrew due to diarrhea	–
Greil et al. 2018	Phase I dose-escalation trial	Cancer patients (≥ 18 y/o)	32	Weekly IV infusions for 8 weeks; 100–300 mg/m^2^	300 mg/m^2^ over 6 h = max tolerated dose; starting dose for trials	Lipocurc™ (liposomal)	Hemolysis in 1, Hb↓ in 3 at 300 mg/m^2^	Pretreated with 50 mg diphenhydramine before each infusion
Hatairaktham et al., 2021	Randomized, controlled study	Non-transfusion-dependent β-thalassemia/Hb E patients	Not specified	500 or 1000 mg/day for 24 weeks	Significant reductions in oxidative stress, inflammation, and hypercoagulability; greater iron reduction at 1000 mg dose	Not mentioned	Not mentioned	Effects varied by baseline ferritin level; higher dose better for iron overload, lower dose more effective for oxidative stress
Javadi et al. 2019	Randomized, double-blind, controlled trial	Rheumatoid arthritis patients	65	40 mg curcumin nanomicelle, 3 times a day for 12 weeks	No significant difference between groups	Not mentioned	Not mentioned	-
Kanai et al. 2012	Dose-escalation, pharmacokinetic study	Healthy adults	6	Single oral dose: 150 mg and after 2 weeks, 210 mg Theracurmin	First nanoparticle curcumin to demonstrate improved bioavailability up to 210 mg	Theracurmin (nanoparticle)	Diarrhea in 1 subject at 150 mg	–
Kanai et al., 2013	Phase I study	Cancer patients	16	200–400 mg curcumin (Theracurmin) daily		Not mentioned	Not mentioned	-
Klickovic et al. 2014	Clinical trial	Healthy males	10	10 g/day for 7 days	Increased HO-1 mRNA expression; effective absorption	Standard curcumin	Not mentioned	–
Lal et al. 1999	Clinical trial	Patients with chronic anterior uveitis	32	375 mg 3 × daily for 12 weeks	Comparable to standard corticosteroid therapy	Standard curcumin	No adverse outcomes observed	Only 18 patients received curcumin alone
Lao et al. 2006	Dose escalation, safety study	Healthy volunteers	24	500–12,000 mg, single dose	Tolerance for single dose up to 12,000 mg excellent	C3 Complex®	30% experienced minimal toxicity	Toxicity did not appear to be dose related
Lopresti et al. 2017	RCT, double-blind, placebo-controlled	Patients with major depressive disorder	123	250 mg or 500 mg curcumin b.i.d. or 250 mg curcumin + 15 mg saffron b.i.d., 12 weeks	All active treatments significantly improved depression and anxiety symptoms vs. placebo	Standard curcumin extract (unspecified brand)	Not mentioned	-
Lopresti et al. 2014	RCT, double-blind, placebo-controlled	Patients with major depressive disorder	56	500 mg twice daily for 8 weeks	Significant improvement in mood symptoms vs. placebo	CurcuWIN	Not mentioned	–
Nakagawa et al. 2014a ), b	Randomized, double-blind, placebo-controlled prospective study	Patients with knee osteoarthritis	25	180 mg/day for 8 weeks	Significant reduction in knee pain VAS; reduced celecoxib dependence	Theracurmin	“No major side effects observed”	No details on side effects
Rasyid et al. 2002	Randomized, single-blind, three-phase, crossover-designed study	Healthy volunteers	12	20–80 mg curcumin for 2 h	Positive cholekinetic effect, dose-dependent	Not mentioned	No adverse outcomes observed	-
Sharma et al. 2001	Dose-escalation pilot study	Patients with colorectal cancer	15	36–180 mg curcumin daily (as 440–2200 mg curcuma extract) for 4 months	Curcuma extract can be administered safely up to 2.2 g/day (180 mg curcumin); curcumin has low oral bioavailability and may undergo intestinal metabolism	Standard curcuma extract	No adverse outcomes observed	–
Storka et al. 2015	Placebo-controlled, double-blind Phase I	Healthy adults	50	Single IV dose: 10–400 mg/m^2^ over 2 h	Safe up to 120 mg/m^2^; higher doses show dose-limiting toxicity signs	Liposomal curcumin	Transient RBC echinocytes ≥ 120 mg/m^2^	Transient MCV increase ≥ 120 mg/m^2^
Sunagawa et al. 2015	Double-blind, 3-way crossover	Healthy adults (5 M, 4 F)	9	Single doses: Theracurmin 182.4 ± 1.0 mg, BCM-95 279.3 ± 10.7 mg, Meriva 152.5 ± 20.3 mg	High absorption efficiency shown for all three formulations	Theracurmin, BCM-95, Meriva	Not mentioned	–
Thota et al. 2018	Cross-over RCT	Healthy adults	16	Single dose: 180 mg CurcuWIN®, with high-carb meal	Significant reduction in postprandial glycemic and insulin response	CurcuWIN® (enhanced)	Not mentioned	Acute study; one-time dose

## Limitations

Results are based on data obtained from advertising websites and the outer packaging of the preparations which limits verification of actual product contents, meaning conclusions may reflect marketing intent rather than true composition or safety, potentially underestimating non-compliance. The data reflect mostly well-marketed products listed on mainstream platforms, underrepresenting informal markets or poorly labeled items. Publicly available data is reliable on manufacturer claims and not independently verified, and there is seldom information concerning quality control, contamination risk, or sourcing integrity. The sample size per country is relatively small, and many of the turmeric supplements did not mention the curcuminoid amount, which skewed the results and limited the ability to draw definitive conclusions. The ranking does not assess the differences in bioavailability. Country regulations differ in supplement labeling, which influences the amount and type of information provided and limits direct comparison across markets. Calculations such as CMDD and SCA rely on the accuracy and completeness of the available data. Any human errors or omissions in the data could impact these calculations and the subsequent analysis. Moreover, this may have led to a better ranking when countries offered less information. Inferential statistics were not used due to the limited sample size, which may not adequately represent the broader market for reliable statistical inference. Future research with larger sample sizes considering the higher bioavailability of certain preparations would be beneficial.

## Conclusion

Turmeric supplements as well as supplement legislation differ significantly internationally. The observed patterns likely result from a combination of consumer preferences, market competition, cultural influences, and regulatory considerations. Additionally, manufacturers may tailor formulations to meet specific regional demands and regulations, marketing strategies, and evolving consumer awareness. In conclusion, the UK offered the safest turmeric supplements overall, particularly offering lower dosages. The USA performed best in naming target audiences, drug interactions, and adverse effects, offering the cheapest products and most up-to-date information, yet it offered the most bioavailable products and lacked sufficient warnings. Germany was overall most transparent in advertising information but also tended to offer more bioavailable products. Indian legislation tended to be the least well enforced, as the supplements performed worst despite similar legal requirements. Australian products offered the least number of bioavailable products, despite offering the most preparations using complexes. Table 12 summarizes country performance in key parameters.

Overall, it appears manufacturers do not often take into account the JEFCA dosage recommendation. This is concerning considering the growing interest in curcuminoid benefits and the increasing research into TEES. The absence of active substance information in many products is concerning, as well as the inadequate warnings for vulnerable demographics. The findings reflect a broader lack of stringent regulation and oversight in the dietary supplement industry. The lack of adherence to expert recommendations prompts consideration of whether supplements should undergo pre-market authorization. Recent research has shown this is not an isolated problem. An analysis of vitamin A supplements (Rathmann and Seifert 2024) as well as gingko supplements (Trabert et al. 2024) highlights similar issues of incompliance regarding labeling requirements as well as questionable safety practices within the supplement industry. Stricter labeling requirements and third-party approval systems would significantly improve safety for consumers. However, based on current literature, it is unclear if taking turmeric supplements present a substantial enough benefit compared to the risks. Further research is required to determine the therapeutic and toxic doses of curcumin through long-term clinical trials, to better understand its safety profile and optimize dosage recommendations.

**Table 12.**
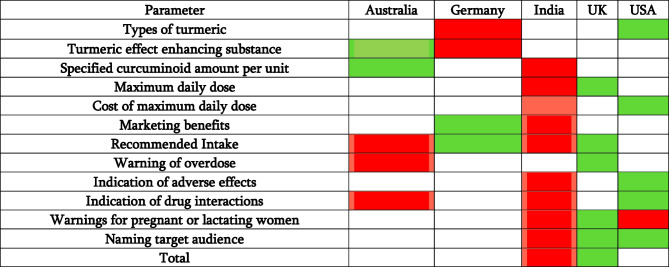
Overview of country comparison

Green indicates best performance, and red indicates worst

## Supplementary Information

Below is the link to the electronic supplementary material.

Supplementary file1 (DOCX 1.72 MB)

## Data Availability

All source data for this work are available upon reasonable request.

## Abbreviations

WHO: World Health Organization
EFSA: European Food Safety Authority
ADI: Acceptable Daily Intake
JECFA: Joint FAO/WHO Expert Committee on Food Additives
FSSAI: Food Safety and Standards Authority of India
TGA: Therapeutic Goods Administration
FDA: Food and Drug Administration
GRAS: Generally Recognized as Safe
CAGR: Compound annual growth rate
CUR: Curcumin
DMC: Demethoxycurcumin
BDMC: Bis-demethoxycurcumin
BfR: German Federal Institute for Risk Assessment
TEES: Turmeric effect enhancing substance
RRP: Regular retail price
SCA: Specified curcuminoid amount
MDD: Maximum daily dose
CMDD: Cost of maximum daily dose
FDA: United States Food and Drug Administration
GMP: Good Manufacturing Practice
EU: European Union
EC: European Commission
FSA: Food Standards Agency
NHC: Nutrition and Health Claims register
CFR: Code of Federal Regulations
FSSR: Food Safety and Standards Regulation, India
GRN: Government Reference Number
aPTT: Activated partial thromboplastin time
PT: Partial thromboplastin time
LD50: Lethal median dose
Nrf2: Nuclear factor erythroid 2-related factor 2
NF-κB: Nuclear factor kappa B
MAPK: Mitogen-Activated Protein Kinase
PI3K/Akt: Phosphoinositide 3-Kinase/Protein Kinase B
DSHEA: Dietary Health and Education Act
