# Supplementary material for: A market and risk assessment of 125 turmeric supplements available in Australia, Germany, India, UK, and USA
Source: Naunyn Schmiedebergs Arch Pharmacol. 2025 Aug 7;399(1):1315–46. doi: 10.1007/s00210-025-04392-5 (PMC12894154; doi:10.1007/s00210-025-04392-5)
Supplement: Supplementary file 1 — Supplementary file1 (DOCX 1.72 MB) [file 210_2025_4392_MOESM1_ESM.docx]

# Supplement

# A Market and risk assessment of 125 Turmeric supplements available in Australia, Germany, India, UK and USA

**Haleema Rahim-Mahdy and Roland Seifert**

**Institute of Pharmacology**

**Hannover Medical School**

**Carl-Neuberg-Straße 1**

**D-30625 Hannover, Germany**

**Corresponding author: Roland Seifert**

**seifert.roland@mh-hannover.de**

**Fig. S1:** Types of turmeric combinations. Analysis of the types of turmeric used in the supplements in each country. The information is presented in a stacked bar chart with each bar showing the total amount of preparations per category. The bar is divided into sections showing the amount of preparations using the corresponding type of turmeric in each country. Each country is represen
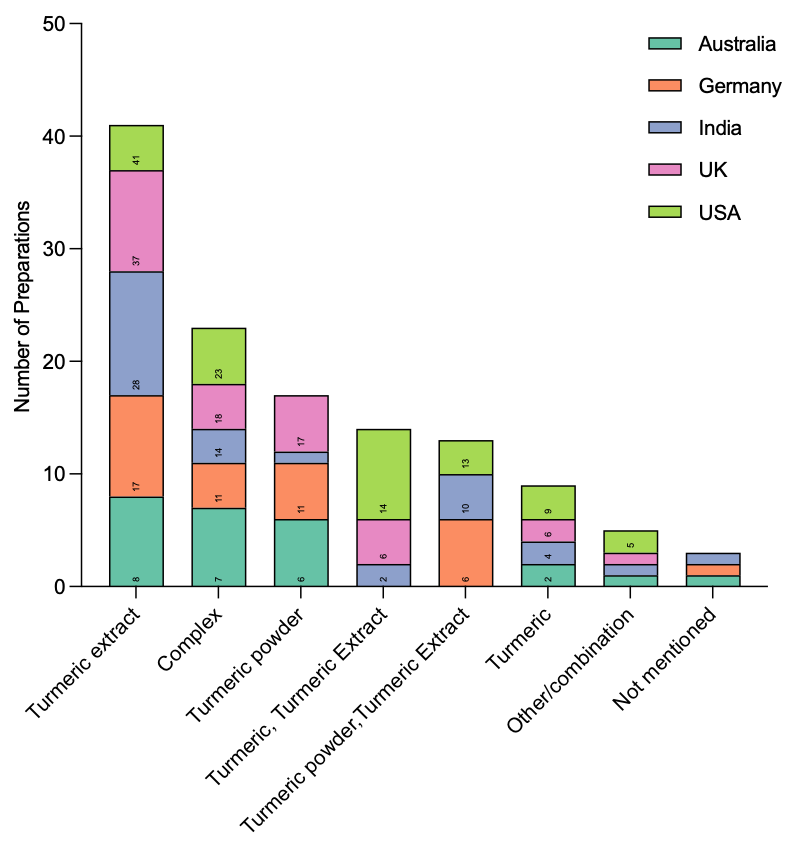
ted by a colour shown in the top right of the diagram. The ‘other/combination' category included 1 product specifying a herbal extract in Australia, 1 product specifying turmeric powder and turmeric concentrate in India, 1 product in the UK specifying the use of turmeric powder, turmeric extract and a complex and 2 products in the USA specifying the use of turmeric powder with a turmeric complex.


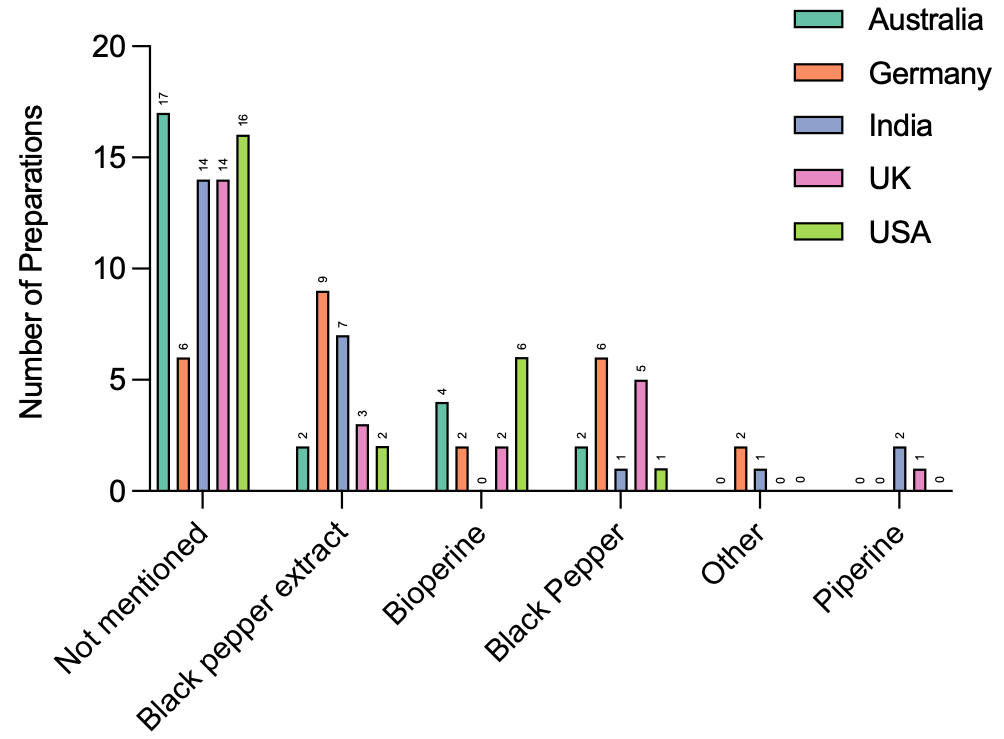


**Fig. S2:** Analysis of the number of preparations using a type of turmeric effect enhancing substance. The information is provided in a grouped column diagram, showing each country in a separate column, identifiable by the color.

**S3 (A)**


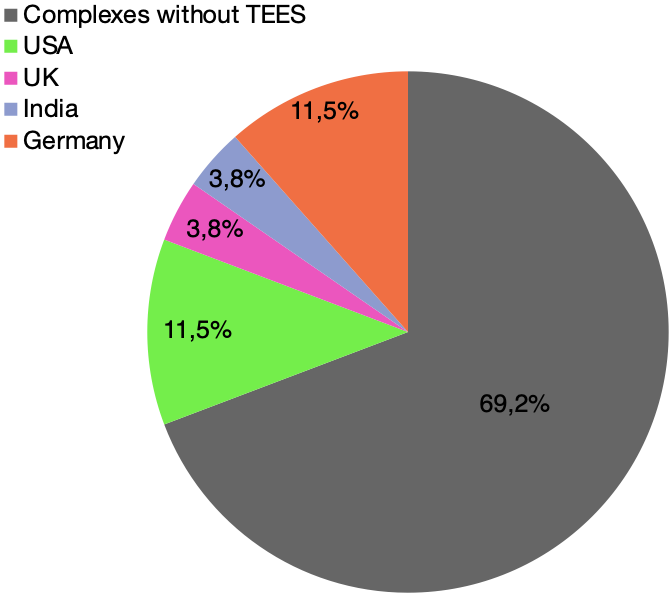


(18)

(3)

(3)

(1)

**S3 (B)
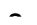
**


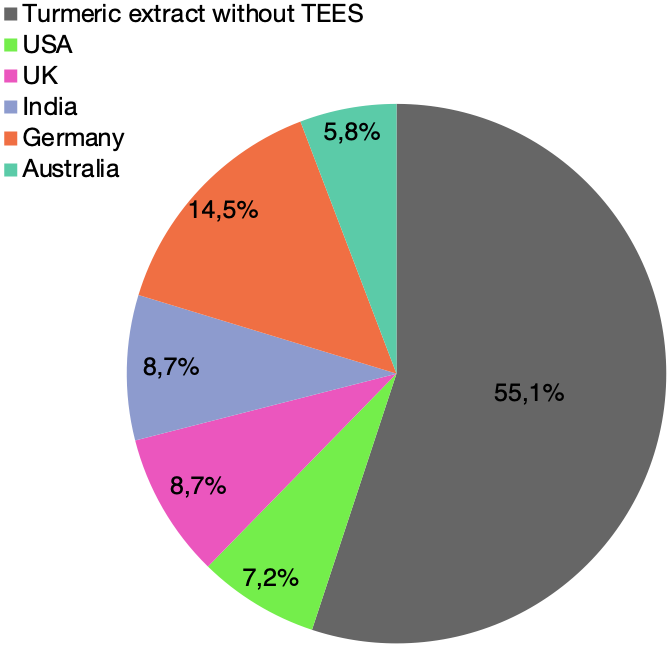


(38)

(6)

(6)

(5)

(10)

(4)


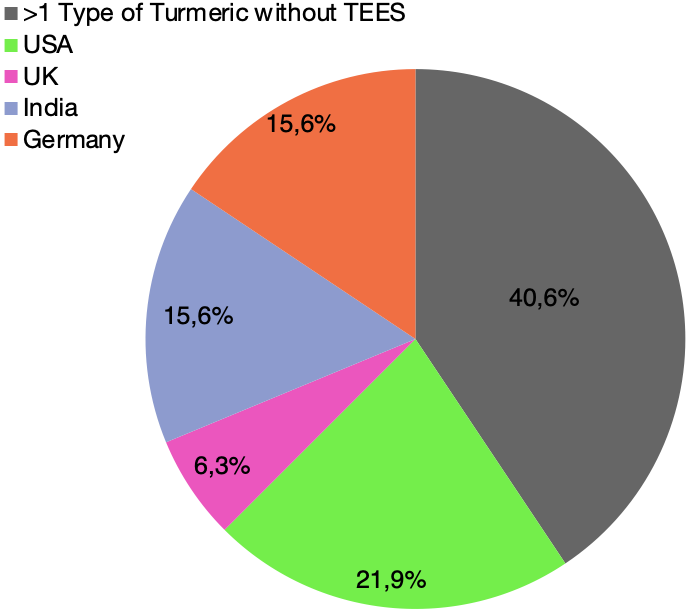


(13)

(7)

(5)

(5)

**S3 (C)**

**Fig. S3 (A):** number of preparations in each country incorporating a complex with and without a TEES

**Fig. S3 (B):** number of preparations in each country using turmeric extract with and without a TEES

(2)

**Fig. S3 (C):** number of preparations in each country combining more than 1 type of turmeric with and without a TEES

Information presented in a pie chart and colour coded for each country.


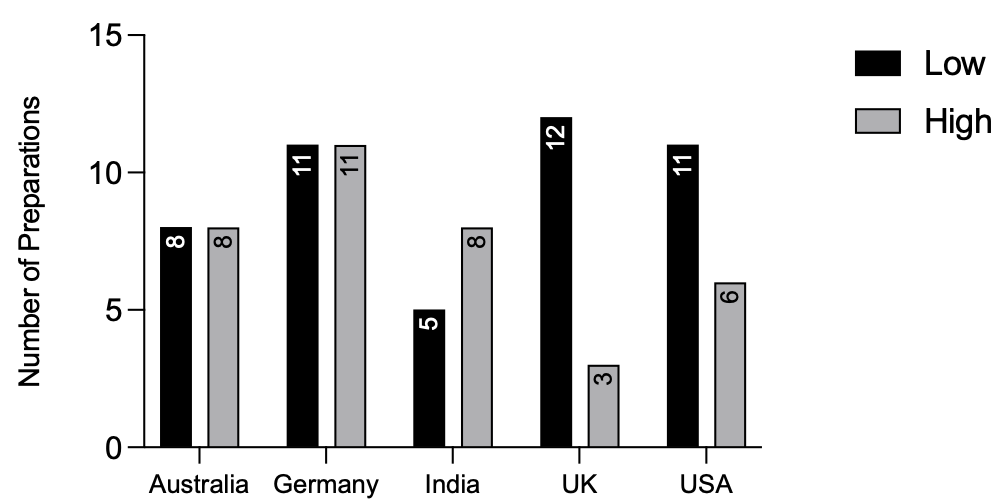
**Fig. S4:** Graphical representation of number of preparations in each country recommending an MDD above and below 210 mg, the maximum JEFCA recommendation for average adult weighing 70 kg (JEFCA, 2004). Information presented in grouped column chart.


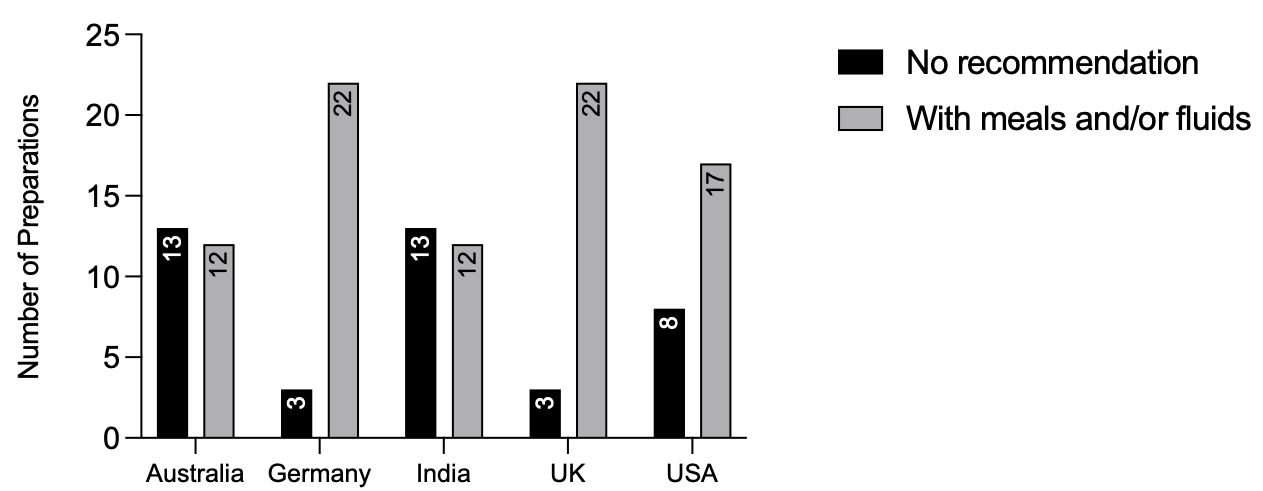

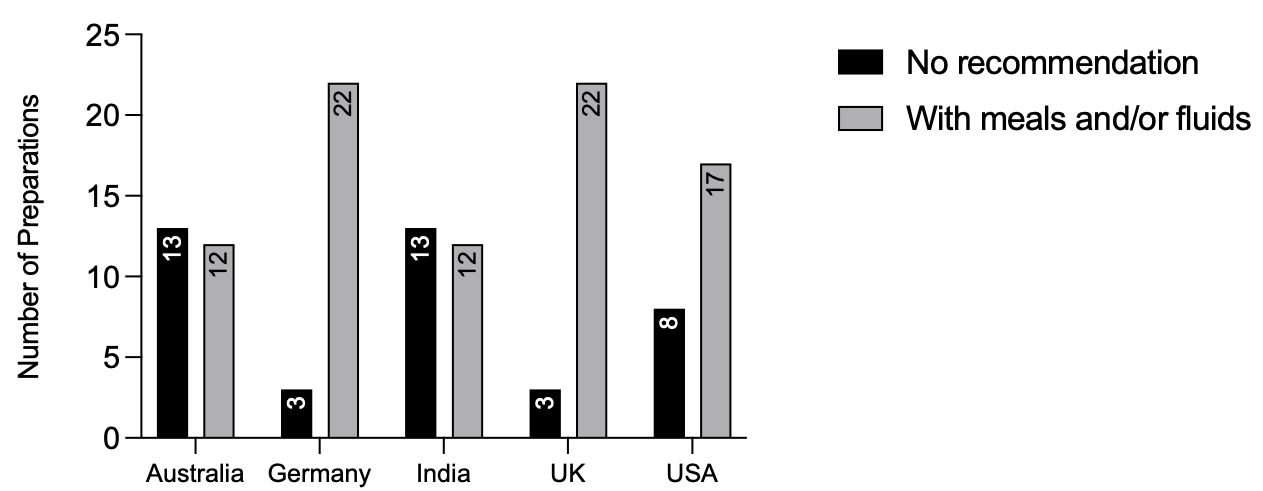


**Fig. S5:** Recommended intake. Graphical representation of number of preparations recommending intake. Information presented in a grouped column chart with black columns showing the number of preparations offering no recommendation and grey columns showing a recommendation with meals and/or fluids


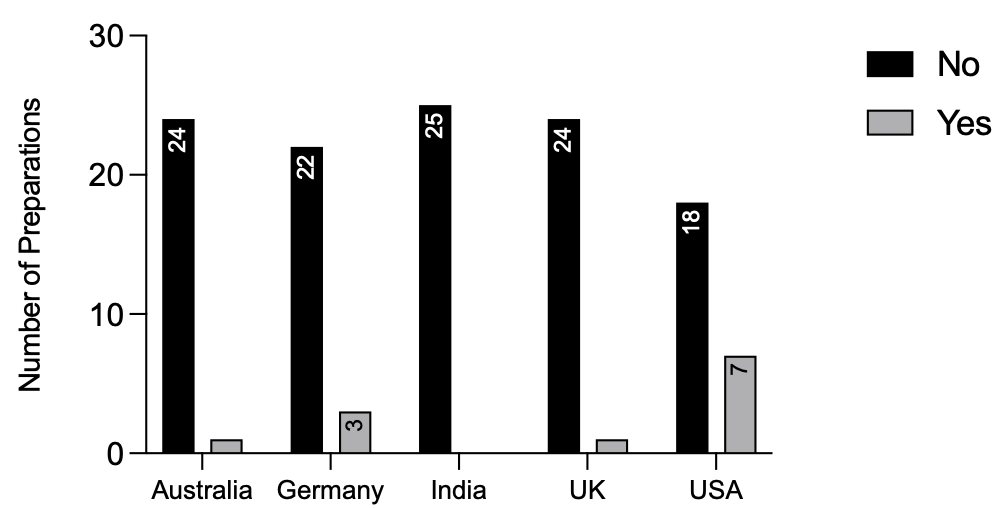

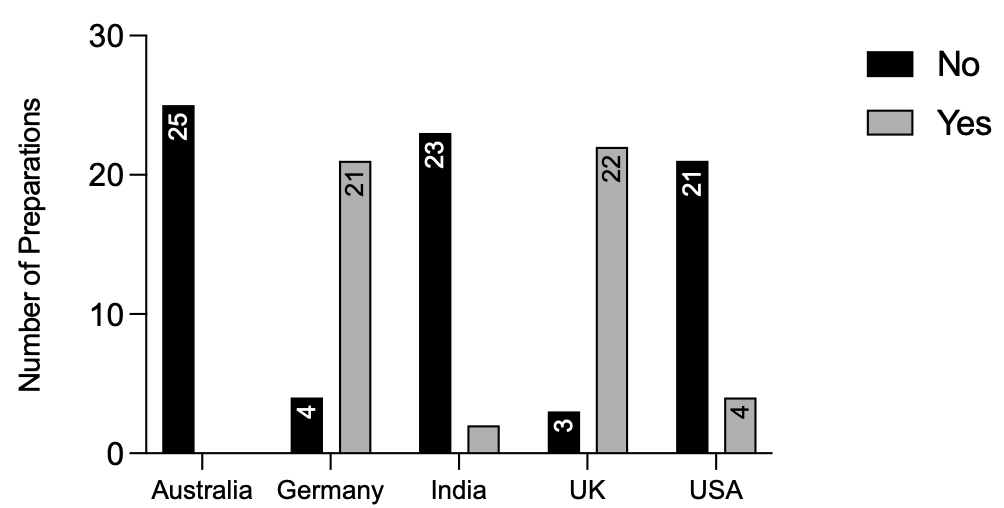
**Fig. S6:** Warning of overdose. Graphical representation of number of preparations offering an overdose warning. Information presented in a grouped column chart, whereby 'yes' is displayed in light grey and 'no' in black.

**Fig. S7:** Indication of adverse effects. Graphical representation of number of preparations indicating any adverse effects. Information presented in a grouped column chart, whereby 'yes' is displayed in light grey and 'no' in black.


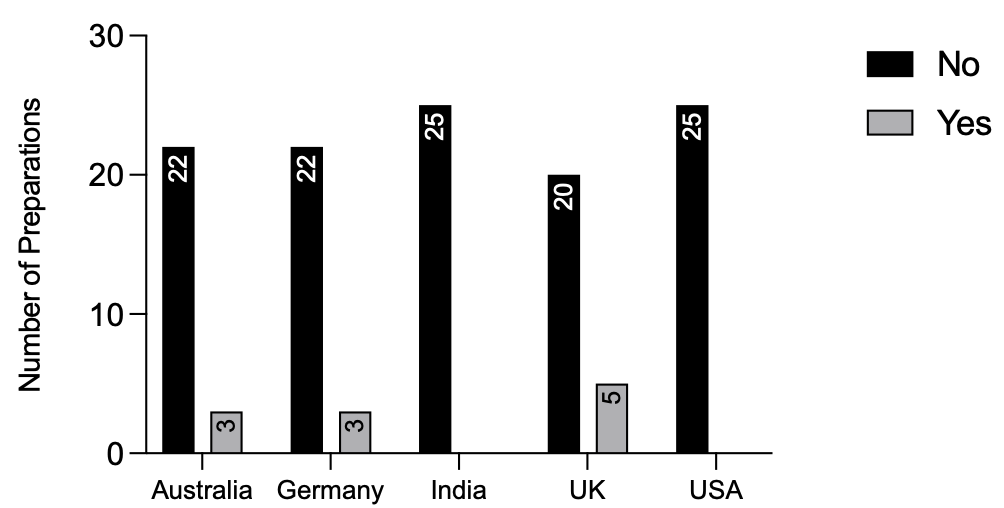

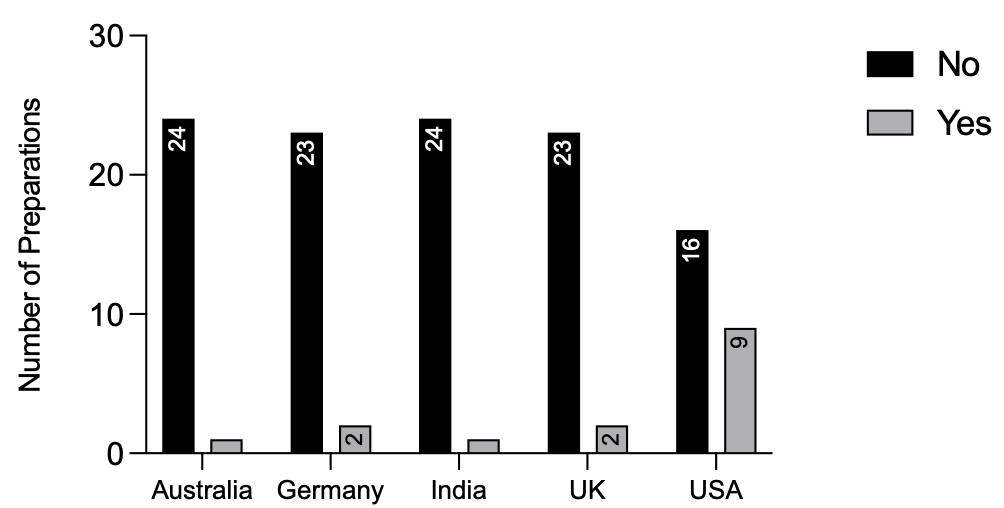
**Fig. S8:** Indication of possible drug interactions. Graphical representation of number of preparations indicating possible drug interactions. Information presented in a grouped column chart, whereby 'yes' is displayed in light grey and 'no' in black.

**Fig. S9:** Warnings for pregnant or lactating women. Graphical representation of number of preparations stating a warning for pregnant or breastfeeding women. Information presented in a grouped column chart, whereby 'yes' is displayed in light grey and 'no' in black.


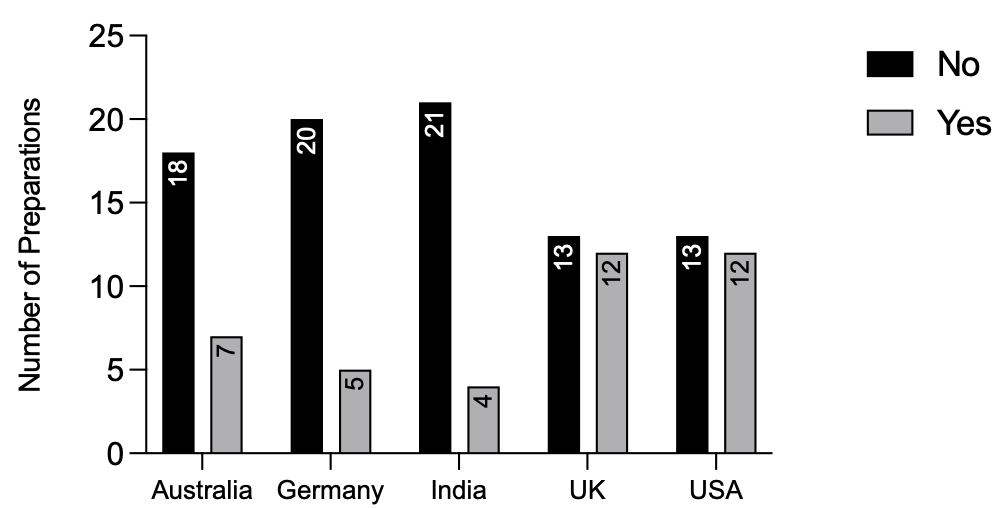


**Fig. S10:** Naming target audiences. Graphical representation of number of preparations naming target audiences. Information presented in a grouped column chart, whereby 'yes' is displayed in light grey and 'no' in black.

**Table S1:**
